# Supplementary material for: Protocol of economic evaluation and equity impact analysis of mHealth and community groups for prevention and control of diabetes in rural Bangladesh in a three-arm cluster randomised controlled trial
Source: BMJ Open. 2018 Aug 20;8(8):e022035. doi: 10.1136/bmjopen-2018-022035 (PMC6104763; doi:10.1136/bmjopen-2018-022035)
Supplement: Author's manuscript [file bmjopen-2018-022035.draft_revisions.pdf]

BMJ Open is committed to open peer review. As part of this commitment we make the peer review history of every article we publish publicly available.

When an article is published we post the peer reviewers' comments and the authors' responses online. We also post the versions of the paper that were used during peer review. These are the versions that the peer review comments apply to.

The versions of the paper that follow are the versions that were submitted during the peer review process. They are not the versions of record or the final published versions. They should not be cited or distributed as the published version of this manuscript.

BMJ Open is an open access journal and the full, final, typeset and author-corrected version of record of the manuscript is available on our site with no access controls, subscription charges or pay-per-view fees (http://bmjopen.bmj.com).

If you have any questions on BMJ Open's open peer review process please email info.bmjopen@bmj.com

Protocol of economic evaluation of m-Health and community groups for prevention and control of diabetes in rural Bangladesh: a three-arm cluster randomised controlled trial

Journal:	*BMJ Open*
Manuscript ID	bmjopen-2018-022035
Article Type:	Protocol
Date Submitted by the Author:	30-Jan-2018

Complete List of Authors:	Haghparast-Bidgoli, Hassan; University College London, Institute for Global Health<br>Shaha, Sanjit ; Diabetic Association of Bangladesh<br>Kuddus, Abdul; Diabetic Association of Bangladesh, Perinatal Care Project<br>Chowdhury, Md.Alimul Reza ; Diabetic Association of Bangladesh<br>Jennings, Hannah ; University College London, Institute for Global Health<br>Ahmed, Naveed; Diabetic Association of Bangladesh<br>Morrison, Joanna; University College London, Institute for Global Health<br>Akter, Kohenour; Diabetic Association of Bangladesh<br>Nahar, Badrun ; Diabetic Association of Bangladesh<br>Nahar, Tasmin ; Diabetic Association of Bangladesh<br>King, C.; UCL, Institute for Global Health<br>Skordis-Worrall, Jolene; University College London Medical School,<br>Batura, Neha; University College London, Institute for Global Health<br>Khan, Jahangir; Liverpool School of Tropical Medicine, Health Economics<br>Mansaray, Anthony ; ICAP Sierra Leone<br>Hunter, Rachael; University College London, Research Dept of Primary Care and Population Health<br>Azad Khan, A.K. ; Diabetic Association of Bangladesh<br>Costello, Anthony ; University College London, Institute for Global Health; World Health Organisation<br>Azad, Kishwar; Diabetic Association of Bangladesh,<br>Fottrell, Edward; University College London, Institute for Global Health

Keywords:	HEALTH ECONOMICS, DIABETES & ENDOCRINOLOGY, Health policy < HEALTH SERVICES ADMINISTRATION & MANAGEMENT

Protocol of economic evaluation of m-Health and community groups for prevention and control of diabetes in rural Bangladesh: a three-arm cluster randomised controlled trial.

Hassan Haghparast-Bidgoli[1*], Sanjit Kumar Shaha[2], Abdul Kuddus[2], Md.Alimul Reza Chowdhury[2], Hannah Jennings[1], Naveed Ahmed[2], Joanna Morrison[1], Kohenour Akter[2], Badrun Nahar[2], Tasmin Nahar[2], Carina King[1], Jolene Skordis[1], Neha Batura[1], Jahangir A. M. Khan[4], Anthony Mansaray[5], Rachael Hunter[6], A.K. Azad Khan[2], Anthony Costello[1, 3], Kishwar Azad[2], Edward Fottrell[1]

1 University College London, Institute for Global Health, London, United Kingdom
2 Diabetic Association of Bangladesh, Dhaka, Bangladesh
3 World Health Organisation, Geneva, Switzerland
4 Liverpool School of Tropical Medicine, Liverpool, United Kingdom
5 ICAP Sierra Leone, Sierra Leone.
6 University College London, Institute of Epidemiology & Health, London, United Kingdom

*Corresponding author
Hassan Haghparast-Bidgoli
Address: University College London, Institute for Global Health, 30 Guilford Street, London WC1N 1EH, UK
Email: h.haghparast-bidgoli@ucl.ac.uk
Tel: +44 (0) 20 7905 2779

Word count: 4173

For peer review only - http://bmjopen.bmj.com/site/about/guidelines.xhtml

Abstract

Introduction: Type 2 Diabetes mellitus (T2DM) is one of the leading causes of death and disability worldwide, generating substantial economic burden for people with diabetes and their families, and to health systems and national economies. Bangladesh has one of the largest numbers of adults with diabetes in the South Asian region. This paper describes the planned economic evaluation of a three-arm cluster randomised control trial of mHealth and community mobilisation interventions to prevent and control T2DM and non-communicable diseases' (NCDs) risk factors in rural Bangladesh (D-Magic trial).

Methods and Analysis: The economic evaluation will be conducted as a within-trial analysis to evaluate the incremental costs and health outcomes of mHealth and community mobilisation interventions compared to the status quo. The analyses will be conducted from a societal perspective, assessing the economic impact for all parties affected by the interventions, including implementing agencies (program costs), health care providers, and participants and their households. Incremental cost-effectiveness ratios (ICERs) will be calculated in terms of cost per case of intermediate hyperglycaemia and T2DM prevented and cost per case of diabetes prevented among individuals with intermediate hyperglycaemia at baseline, and cost-per mm Hg reduction in systolic blood pressure. In addition to ICERs, the economic evaluation will be presented as a cost-consequence analysis where the incremental costs and all statistically significant outcomes will be listed separately. Robustness of the results will be assessed through sensitivity analyses.

Ethics and Dissemination: The approval to conduct the study was obtained by the University College London Research Ethics Committee (4766/002) and by the Ethical Review Committee of the Diabetic Association of Bangladesh (BADAS-ERC/EC/t5100246). The findings of this study will be disseminated through different means within academia and the wider policy sphere.

Trial registration: ISRCTN41083256. Registered 30/03/2016

For peer review only

For peer review only - http://bmjopen.bmj.com/site/about/guidelines.xhtml

Strengths and limitations of this study

- This protocol paper reports planned data collection and analyses alongside a complex public health trial to ensure transparency.
- The findings of this study will provide valuable evidence for decision-makers when considering the potential implementation of novel mHealth and community mobilisation through a Participatory Learning and Action Approach interventions for the prevention of diabetes and mitigation of NCDs risk factors
- The applicability of the findings may be limited to similar settings, and the interventions may require further adaptation to ensure feasibility and sustainability in similar or other contexts.

For peer review only

BACKGROUND

Burden of diabetes mellitus

Diabetes mellitus, mainly type 2, is a one of the leading causes of death and disability worldwide. It is estimated that around 415 million people worldwide, or 9% of adults aged 20-79 have diabetes, with about 75% of people living with diabetes residing in low- and middle-income countries (LMICs) (1). It is predicted that by 2040, one in 10 adults will have diabetes, unless preventive efforts are undertaken (1). Diabetes, if not managed properly, can lead to several complications, such as heart attack, kidney failure, leg amputation, vision loss and several other long-term consequences, that impact significantly on quality of life and cause premature death (1, 2). Diabetes and its complications create a substantial economic burden for people with diabetes and their families, and for health systems and national economies through direct medical costs and productivity loss (2).

Bangladesh has the second largest number of adults with diabetes in the South Asian region, with around 7 million adults aged 20-79 years with diabetes (1). The prevalence of diabetes among adults (20-79 years old) in Bangladesh is estimated to be around 8.5 % (1, 2), with a three to four-fold increase since the 1990s (3, 4). It is predicted that prevalence of diabetes in Bangladesh will reach 23.6% in men and 33.5% in women by 2030, unless preventive efforts are undertaken (3).

Underlying the increasing prevalence of diabetes, globally and in Bangladesh, are complex genetic, environmental and lifestyle factors, including changes in dietary habits and increases in risk factors like smoking and physical inactivity (2). Effective interventions are available to prevent type 2 Diabetes mellitus (T2DM), and to prevent the complications and premature death from diabetes (2). The evidence from LMICs shows that lifestyle and other non-pharmacological interventions can prevent and delay the onset of T2DM and its complications (5). However, there is a lack of cost effective programs designed specifically for Bangladeshi populations, taking into account their contextual needs and resources.

The Bangladesh D-Magic Trial

The Bangladesh D-magic is a three-arm cluster randomised control trial (cRCT) of mHealth and community mobilisation interventions, conducted in four rural upazilas in Faridpur district, Bangladesh. The trial aims to assess the effectiveness and cost-effectiveness of the interventions in the prevention and control of T2DM and NCD risk factors in rural Bangladesh. In the D-magic trial, the clusters (96 villages) were randomised to receive either the mHealth intervention, the community mobilisation intervention, or be in the control arm (32 in each). In the mHealth intervention, individuals receive voice messages about prevention and control of NCD risk factors and T2DM on their mobile phone. In the community mobilisation intervention, a trained facilitator initiates a series of diabetes and NCD risk-factor focused monthly group meetings for men and women, working through a participatory learning and action (PLA) cycle by which group members themselves identify, prioritise and tackle

For peer review only - http://bmjopen.bmj.com/site/about/guidelines.xhtml

problems associated with T2DM and its risk factors. In addition, all study areas (both intervention and control) clusters receive health system strengthening activities, which include the training/refresher training of health care workers working in the community and health facilities, in the prevention, diagnosis and treatment of T2DM, as well as the development of essential equipment inventories.

Details of the D-Magic trial are described in detail elsewhere (6). This protocol paper aims to fully describe the methodology for the economic evaluation of the trial.

Economic evaluations of community and mHealth interventions for prevention and control of diabetes

There is evidence that mHealth programs can have a positive impact on behaviour change and prevention and control of diabetes and NCDs in high-risk populations (7-10). However, there is little information on the cost and cost-effectiveness of mHealth interventions for the prevention and control of NCDs (11-13). Two recent systematic reviews of the economic evidence of mHealth (12), and mHealth for diabetes prevention and control (13), have shown that there are a handful of NCD and diabetes interventions that have reported cost and cost effectiveness evidence. Nearly all of these studies have been conducted in high-income settings. The majority of these studies report that mHealth interventions are cost-effective or cost saving, though the quality of reported evidence was not satisfactory in some of cases (12, 13).

Similarly, although there is some evidence on effectiveness of community-based interventions in the management of T2DM in low-income settings (14-18), there is little evidence on how cost effective these interventions might be (13). A recent review (13) has identified 10 community-based interventions on preventing and controlling diabetes. These interventions, which are largely implemented in high-income settings, have reported that community-based interventions are cost-effective or cost-saving approaches in the management of T2DM (13).

The current study will be the first to assess the cost-effectiveness of community mobilisation through PLA in the prevention of T2DM. This approach has previously been shown to be highly cost-effective in improving maternal and newborn health (19, 20). This study will also contribute to the evidence on cost-effectiveness of mHealth interventions for preventing T2DM in LMIC settings.

Aim and objectives

The D-Magic economic evaluation aims to measure the cost-effectiveness of mHealth and participatory community group interventions to prevent and to control T2DM and NCD risk factors in rural Bangladesh from a societal perspective.

The specific objectives of the D-Magic economic evaluation are:

1. To estimate the costs of setting up and implementing the mHealth and participatory community group interventions as well as health system strengthening (HSS) activities;

For peer review only - http://bmjopen.bmj.com/site/about/guidelines.xhtml

2. To calculate the costs to the healthcare system, of increased care-seeking (i.e. diagnosis and treatment) for T2DM and other NCDs, as a result of the D-Magic interventions;
3. To measure costs associated to the intervention participants and their households of changes in diabetes or other NCD-related diagnosis and management care-seeking costs as well as any costs associate with changes in diet and other life-style behaviours, as a result of the D-Magic interventions;
4. To present the incremental costs and outcomes of the interventions as a cost-consequence analysis;
5. To calculate the incremental cost-effectiveness of the mHealth and community mobilisation interventions combined with HSS activities, as compared to HSS activities alone, where all new HSS activities are delivered in addition to the existing government programmes.

METHODS

Study setting and population

The study setting for the D-Magic trial is Faridpur district, located south of Dhaka. The district has a population of over 1.7 million people in a high-density area of just over 2000 square kilometres. It is primarily an agricultural economy, with the main crops being jute and rice. Like in the rest of Bangladesh, healthcare is provided at three levels: primary care is provided at Community Clinics and at Union Health and Family Welfare Centres; secondary level care providers (both in- and out-patient services) are sub-district (Upazilla) health complexes and hospitals; and tertiary care is provided at district hospitals and medical college hospitals (21). Private and informal service providers are also present in the area, with the informal sector being the main provider in rural areas (21), Faridpur district included. Inadequate and inequitable access to services, shortages of skilled health care providers, short supplies of medicines and poor quality and low responsiveness of services are the main challenges faced by the Bangladesh healthcare system (21), and remains a challenge in Faridpur district too.

The study population for the D-Magic study are men and non-pregnant women who are aged 30 years or more and permanent residents (i.e. lived there for more than 6 month) of 96 villages (clusters) in four rural upazillas – Nagarkanda, Boalmari, Saltha and Madhukhali – in Faridpur district.

Trial design

The D-Magic trial is a three-arm cRCT which is implemented in four rural upazilas in Faridpur district, Bangladesh. In D-Magic trial, 96 clusters (villages) were randomised to receive either the mHealth intervention, the community mobilisation intervention, or be in the control arm. All three arms receive a number of health system strengthening activities. The interventions will be completed by end of December and all data collection will be ongoing until May 2018. A brief description of the mHealth, community mobilisation and health system strengthening

For peer review only - http://bmjopen.bmj.com/site/about/guidelines.xhtml

interventions are presented in the following sections; a full description of the interventions is presented elsewhere (6).

mHealth intervention

The mHealth intervention involves free of charge voice messages about the prevention and control of T2DM and NCD risk factors sent twice-weekly to the individual's mobile phone during an 18-month period. The intervention and the messages' content were developed based on findings from baseline formative research in the study area, and application of a number of behaviour change theories, such as the COM-B (Capability, Opportunity, Motivation, Behaviour) theory for understanding behaviour (22) and the Theoretical Domains Framework (TDF) to encourage change (23, 24). The mHealth intervention is available to all individuals who have access to a mobile phone and registered to receive the messages by providing their mobile number to the intervention community recruiters.

Community Mobilisation intervention

The community mobilisation intervention involves initiation and facilitation of separate male and female participatory groups, with approximately 20 members in each. The intervention is an adaptation of a participatory women's groups intervention implemented in South Asia (including Bangladesh) and Sub-Saharan African settings and shown to be effective and cost effective at reducing neonatal mortality (20, 25). The groups progress through a series of 18 monthly meetings following the four phases of PLA. During phase 1, participants identify and prioritise factors that affect their health, particularly those increasing their risk of developing or failing to manage T2DM; in phase 2, the participants and their community come up with feasible strategies that can be implemented to address the problems identified in phase 1; during phase 3, they implement these strategies; in phase 4 they evaluate the strategies they have implemented. The groups are run by salaried facilitators, who are recruited from the study areas and have a minimum of higher secondary school education. The facilitators undertook one week's training on group facilitation and basic health messages related to NCD prevention and control, in particular T2DM. They also have received refresher training during the course of the interventions.

Health Systems Strengthening (HSS)

A series of health system strengthening activities are carried out in all study areas (both intervention and control clusters). These activities, which are tailored according to the mapping of health care providers in the project area in the formative phase of the project, included the training of mainly informal health care workers in the community and distributing educational materials among formal and informal providers in prevention, diagnosis and treatment of T2DM, as well as the development of essential equipment inventories.

Measurement of health outcomes/effectiveness

For peer review only - http://bmjopen.bmj.com/site/about/guidelines.xhtml

The D-Magic trial will test the effect of the community mobilisation intervention and mHealth intervention relative to the control and does not directly compare the effects of each intervention relative to the other. Analysis of the outcomes will be by intention to treat at the individual and cluster level as appropriate. Moreover, participants with missing data on the primary outcomes will be excluded from primary outcome analysis (6).

Primary outcome

The D-Magic trial has two primary outcomes: (1) combined prevalence of intermediate hyperglycaemia (i.e. impaired fasting glucose or impaired glucose tolerance) and T2DM, and (2) cumulative two-year incidence of T2DM among individuals identified with intermediate hyperglycaemia at the start of the trial (6). Using the prevalence data, the number of intermediate hyperglycaemia and diabetes mellitus cases prevented, and the number of diabetes cases prevented among individuals with intermediate hyperglycaemia at baseline will be calculated as the difference between the expected and the actual number of cases using the adjusted odds ratio relative to the control population.

Secondary outcomes

The trial has a number of secondary outcomes including diastolic and systolic blood pressure, prevalence of hypertension, body mass index, prevalence of overweight and obesity, prevalence of abdominal obesity, health-related quality of life, and psychological distress among self-reported diabetics (6).

Comparison will be made between the interventions (mHealth and community mobilisation) and control (HSS activities only) to estimate incremental cost-effectiveness ratios (ICERs) for the primary outcomes; in terms of cost per case of intermediate hyperglycaemia and diabetes mellitus prevented and cost per case of diabetes prevented among individuals with intermediate hyperglycaemia at baseline, and for some of the secondary outcomes such as cost- per mm Hg reduction in systolic BP. ICER will be conducted if a significant impact on the outcomes is observed. Moreover, all costs and (statistically significant) outcomes, both primary and secondary, will be presented separately in a cost-consequence analysis.

Willingness to pay/Contingent Valuation study

We will conduct a willingness to pay **(**WTP) study in order to elicit maximum monthly amount of money each participant or household would be willing to pay if an m-Health service on diabetes and NCD risk factors' prevention and management (i.e. weekly voice messages) was available.

WTP studies are widely used in order to elicit monetary value of a service or good not available in the market (26-29). In the Bangladesh context, Islam et al (2015) in a recent study estimated WTP of T2DM patients for receiving messages for increasing adherence to treatment (30). Similar to Islam et al (2015), we will use an open-ended contingent valuation (CV) method (26, 29, 31), asking participants through an open-ended question how much they would be willing to pay monthly to receive voice

For peer review only - http://bmjopen.bmj.com/site/about/guidelines.xhtml

messages related to diabetes and NCD risk factors' prevention and management, if such a service was available. Open-ended CV is a more flexible approach and avoids starting point bias or range bias introduced by other methods such bidding game and payment cards (26). The theoretical framework defined by O'Brien and Gafni (27) for CV studies will be used to design the study.

WTP questions will be asked from all the participants at the end-line impact evaluation survey. WTP values will be compared across different groups, for example, based on exposure to the interventions (i.e. among those exposed to mHealth, or exposed to participatory groups, and participants who have not been exposed to either of these interventions) or health condition (people diagnosed with diabetes or other NCDs and others). In addition, for each participant, detailed individual and household level socio-economic characteristics will be collected to examine the extent to which WTP values will vary by socio-economic status of participants.

Equity impact of the D-Magic interventions

In addition to measuring efficiency/cost-effectiveness of the D-Magic interventions, an analysis of equity impact of the interventions will be conducted to assesse whether impacts/gains from the intervention are equitably shared among the target population. This will be done through sub-group analyses of the primary and secondary outcomes based on the socio-economic status of the target population.

Identification, measurement and valuation of resource use

The cost-effectiveness and cost-consequence of the D-Magic interventions will be measured from a societal perspective (32, 33); measuring the economic impact for all parties affected by the interventions, including implementing agency (or program costs), public healthcare providers (at both local and national levels) and users, who are the intervention participants and their households. The following sections provide a detailed description of the proposed methods for measuring and valuing programme costs, healthcare provider costs and participants/household costs. Programme costs include those incurred by the implementing agency or program provider i.e. Diabetic Association of Bangladesh (BADAS). The healthcare provider costs are those incurred by the government health facilities in the study area including community clinics, Health & Family Welfare centres and Upazila Health Complex/hospitals. The household or user costs include those incurred by participants and their households.

Table 1 provides an overview of the financial and economic costs to be employed in the economic evaluation of the D-Magic interventions.

Programme-related costs

Direct and indirect costs of designing and implementing mHealth, community mobilisation and health system strengthening interventions will be estimated using a combination of activity-based costing (34) and ingredients approach (35).

Financial or expenditure data

For peer review only - http://bmjopen.bmj.com/site/about/guidelines.xhtml

Programme costs are mainly financial or accounting costs, which are collected prospectively from the project accounts or expenditure records of the implementing partner and entered (generally, on an annual basis) to an MS Excel data capture tool. The tool contains different sections/worksheets that will allow the categorization of costs into line items (i.e. staff, materials, capital, and joint costs), start up and implementation costs of the interventions, and costs associated with the different programme components, i.e. m-Health, community mobilization, HSS, and monitoring and evaluation. Key informant interviews with project leads and monthly/quarterly staff time sheets will be used to allocate joint costs between the programme components. The summary worksheets in the cost data capture tool present the costs by programme component (e.g. mHealth, community mobilisation and HSS), summarise the total cost data per intervention, allows effect data to be entered and estimates the cost-effectiveness results.

Donated items and opportunity costs

Some items, such as donated items and volunteer time, are not captured in the accounting system and need to be converted to economic costs using their market value and then entered into the data capture tool (36-38). Potential donated items are equipment donated by the implementing agency (i.e. purchased by previous projects and used in the D-Magic project). The donated items will be identified through key informant interviews with the project leads.

The majority of volunteer time is related to designing messages for the mHealth intervention, where several meetings were held with experts who were volunteers. Detailed information regarding these meetings, including the number of meetings, their duration, and the participants is being documented by the project. The opportunity cost of the time invested by the experts will be measured as a proportion of their salary or a salary equivalent using published national/local wage rate reports.

Public healthcare providers costs

The D-Magic project is likely (at least in the short term) to increase seeking care for diabetes, other NCDs and NCD risk factors such as hypertension, particularly demand for services such as testing and treatment for hypertension, diabetes and pre-diabetes, or seeking advice or treatment for weight control. In addition, there is a time (opportunity) cost of direct involvement in the HSS activities for the healthcare providers (Table 1).

Cost of changes in demand for services

The costs to public healthcare providers in the project area due to increased (or any changes in) demand for their services will be estimated. A mapping of the health care providers in the study area has been completed and 20 functional governmental health care facilities, at different levels, have been identified. These facilities included 14 Community Clinics, 3 Health & Family Welfare centres and 3 Upazila Health Complex/Hospitals. A sample of 11 health facilities including 8 community clinics and 2 Health & Family Welfare centres (with equal numbers in control and

For peer review only - http://bmjopen.bmj.com/site/about/guidelines.xhtml

intervention clusters), as well as one Upazila Health Complex/Hospital (which cover both control and intervention areas) were selected for baseline audits of diabetes and NCD services, estimating their resource utilisations and unit costs. Similar audits and cost data collection will be conducted for the same facilities post-intervention in order to assess the changes in NCD service utilisation attributable to the D-Magic interventions. This data will be complemented by health seeking behaviour information collected from the study participants in control and interventions clusters at the D-Magic end-line impact evaluation survey. Differences in service utilisation between intervention and control areas will be attributed to the D-Magic interventions.

A simple audit and cost-capture tool was developed for facility data collection and piloted with facilities at different levels. Data from the cost-capture tool will be complemented by the existing data from the facility reports, and published data. Costs of services provided by the facilities will be estimated using a step-down approach (39).

Any change in demand for services provided in the facilities other than those mentioned above and the services not covered by them, in intervention areas compared to the control, will be identified during the trial's routine monitoring and end-line impact evaluation survey. Any cost of that change in demand will be calculated using published data on the unit costs of those services.

Opportunity cost of HSS activities

Moreover, as discussed earlier, HSS activities include several training sessions for health care providers in prevention, diagnosis and treatment of T2DM. Information on the number of meetings, their duration and participation is being documented by the project. The opportunity cost of the time spent by the providers will be measured as a proportion of their salary for formal providers or as a salary equivalent, for informal providers.

Participants and their household costs

D-Magic interventions may influence participants and their households' costs in a number of ways. These include changes in health seeking behaviour, and changes in household lifestyles that might affect food and non-food consumption patterns and spending as well as time spent engaging in physical activities. It also includes the time (opportunity) cost of participation in the PLA group meetings and participating in the actions taken by the groups (Table 1).

Health care seeking costs

D-Magic may increase the participants and their households' seeking advice and care from both formal and informal providers for testing and treatment for hypertension, diabetes, pre-diabetes and other NCDs. Information on costs of care seeking are collected from all the participants recruited in the project, at the baseline and end-line evaluation surveys. This information will be complemented by the data collected from the household consumption expenditure survey. The difference in spending for the

For peer review only - http://bmjopen.bmj.com/site/about/guidelines.xhtml

participants and their households will be calculated and compared between intervention and control areas.

Changes in household food and non-food expenditure

Changes in food and non-food expenditure will be captured in a comprehensive household consumption and expenditure survey. The survey will be conducted on a random sub-sample of 300 households (100 per trial arm) at the end of intervention period. The changes in the expenditure will be compared between interventions and control areas.

Opportunity cost of participation in the interventions

Participating in the PLA group meetings incur some costs to the participants and their family. These costs include the direct costs (e.g. cost of getting to the group) and time cost of group participation (e.g. travel time and time spent in the group) and participating in the actions taken by the group, or changing health and lifestyle behaviours. Information on the potential direct and time costs will be collected through a sub-sample survey of 312 group participants (both male and female), randomly selected. Sample size for the survey was primarily calculated to give sufficiently accurate estimate of group participants' characteristics.

Cost-effectiveness and cost-consequence analyses

Economic evaluation will be conducted as a within-trial analysis using the intention-to-treat results, and will be presented in terms of ICERs, calculated as the difference in total costs of mHealth and community mobilisation interventions (plus HSS activities) versus HSS activities only (or control), divided by the difference in mean effects of each interventions versus control (40) (41). As mentioned previously, ICERs will be evaluated in terms of cost per case of intermediate hyperglycaemia and T2DM prevented and cost per case of T2DM prevented among individuals with intermediate hyperglycaemia at baseline, and for some of the secondary outcomes such as cost- per mm Hg reduction in systolic BP. In addition to ICERs, the economic evaluation will be presented as a cost-consequence analysis where the incremental costs and all statistically significant outcomes will be listed separately, allowing policymakers to compare the costs and all impacts/gains of the D-Magic interventions. Cost-consequence analysis has been recommended for complex public health interventions, such as D-Magic, that have multiple health and non-health impacts, which are difficult to measure in a common outcome unit (41, 42).

All costs will be presented in 2017 prices in Bangladeshi Taka and International Dollars (INT$). All costs will be adjusted for inflation using the Bangladeshi Consumer Price Index (CPI) and will be converted to 2017 INT$ using the Purchasing Power Parity (PPP) conversion factor for Bangladesh. Moreover, both costs and outcomes will be discounted using a standard discount rate of 3%, as recommended by WHO-CHOICE (43) and the Gates Reference Case for Economic Evaluation (44). The impact of uncertainty in key parameters on the cost-effectiveness results will be assessed through a series of deterministic and

For peer review only - http://bmjopen.bmj.com/site/about/guidelines.xhtml

probabilistic sensitivity analyses. Reporting of the study design, analytical methods and findings will follow the Consolidated Health Economic Evaluation Reporting Standards (CHEERS) statement (45). The D-Magic interventions will be judged to be cost-effective and affordable (though, indirectly) against the WHO-CHOICE recommendation (43), as well as recently developed cost-effectiveness thresholds (46).

The possibility of conducting an extended cost-effectiveness analysis (47) alongside modeling national scale up of the D-Magic interventions will be explored. Moreover, we will explore the possibility of running a long-term cost-effectiveness analysis using decision analytical modelling based on the relevant outcomes such as systolic blood pressure, body mass index, etc. (if statistically significant) to predict future economic impacts from implementing the D-Magic interventions on the target population.

CONCLUSION

In order to increase transparency and minimise bias, publication and peer-review of economic evaluation protocols is encouraged. The economic evaluation of the D-Magic interventions will contribute to the scarce cost-effectiveness evidence on mHealth and community mobilisation interventions for preventing diabetes and NCDs. The findings from this study will provide decision-makers in Bangladesh and other similar low-resource settings with valuable information to inform any future implementation of these innovative interventions.

Contributors

HHB, EF, AK, HJ, JM, CK, JS, NB, JK, RH, AKAK, AC and KA contributed to the study design; HHB, EF, CK, JM contributed to the statistical analyses; SKS, MARC, NA, KA, BN and TN contributed to data acquisition; HHB was responsible for the initial drafting of this manuscript; all authors contributed to the review of this manuscript and provided comments. All authors read and approved the final manuscript.

Funding

The trial is funded by the Medical Research Council UK (MR/M016501/1) under the Global Alliance for Chronic Diseases (GACD) Diabetes Programme.

Competing interests

The authors declare they have no competing interests.

Ethics approval

The D-Magic trial has been approved by the University College London Research Ethics Committee (4766/002) and by the Ethical Review Committee of the Diabetic Association of Bangladesh (BADAS-ERC/EC/t5100246). The trial has been

For peer review only - http://bmjopen.bmj.com/site/about/guidelines.xhtml

registered and assigned an International Standard Randomised Controlled Trial Number (ISRCTN41083256).

References

1. International Diabetes Federation. Diabetes Atlas: Seventh Edition. Brussels, Belgium: International Diabetes Federation; 2015.
2. World Health Organization. Global report on diabetes. Geneva, Switzerland World Health Organization; 2016.
3. Rahman MM, Akter S, Jung J, Rahman MS, Sultana P. Trend, projection, and appropriate body mass index cut-off point for diabetes and hypertension in Bangladesh. Diabetes research and clinical practice. 2017;126:43-53.
4. Saquib N, Saquib J, Ahmed T, Khanam MA, Cullen MR. Cardiovascular diseases and type 2 diabetes in Bangladesh: a systematic review and meta-analysis of studies between 1995 and 2010. BMC public health. 2012;12:434.
5. Rawal LB, Tapp RJ, Williams ED, Chan C, Yasin S, Oldenburg B. Prevention of type 2 diabetes and its complications in developing countries: a review. International journal of behavioral medicine. 2012;19(2):121-33.
6. Fottrell E, Jennings H, Kuddus A, Ahmed N, Morrison J, Akter K, et al. The effect of community groups and mobile phone messages on the prevention and control of diabetes in rural Bangladesh: study protocol for a three-arm cluster randomised controlled trial. Trials. 2016;17(1):600.
7. Hamine S, Gerth-Guyette E, Faulx D, Green BB, Ginsburg AS. Impact of mHealth chronic disease management on treatment adherence and patient outcomes: a systematic review. Journal of medical Internet research. 2015;17(2):e52.
8. Shaw R, Bosworth H. Short message service (SMS) text messaging as an intervention medium for weight loss: A literature review. Health informatics journal. 2012;18(4):235-50.
9. Shetty AS, Chamukuttan S, Nanditha A, Raj RK, Ramachandran A. Reinforcement of adherence to prescription recommendations in Asian Indian diabetes patients using short message service (SMS)--a pilot study. The Journal of the Association of Physicians of India. 2011;59:711-4.
10. Ramachandran A, Snehalatha C, Ram J, Selvam S, Simon M, Nanditha A, et al. Effectiveness of mobile phone messaging in prevention of type 2 diabetes by lifestyle modification in men in India: a prospective, parallel-group, randomised controlled trial. The lancet Diabetes & endocrinology. 2013;1(3):191-8.
11. Stephani V OD, Quentin W,. Does mHealth contribute to improved care for people with non-communicable diseases in developing countries? A systematic review of randomized controlled trials. Berlin: Universitätsverlag der TU Berlin; 2015.
12. Iribarren SJ, Cato K, Falzon L, Stone PW. What is the economic evidence for mHealth? A systematic review of economic evaluations of mHealth solutions. PloS one. 2017;12(2):e0170581.
13. Mansaray A, Haghparast-Bidgoli H. Economic evidence on mHealth and community mobilisation interventions for the prevention and management of type 2 diabtes: A systematic review. 2017.
14. Balagopal P, Kamalamma N, Patel TG, Misra R. A community-based diabetes prevention and management education program in a rural village in India. Diabetes care. 2008;31(6):1097-104.

For peer review only - http://bmjopen.bmj.com/site/about/guidelines.xhtml

15. Balagopal P, Kamalamma N, Patel TG, Misra R. A community-based participatory diabetes prevention and management intervention in rural India using community health workers. The Diabetes educator. 2012;38(6):822-34.
16. Sathish T, Williams ED, Pasricha N, Absetz P, Lorgelly P, Wolfe R, et al. Cluster randomised controlled trial of a peer-led lifestyle intervention program: study protocol for the Kerala diabetes prevention program. BMC public health. 2013;13:1035.
17. Assah FK, Atanga EN, Enoru S, Sobngwi E, Mbanya JC. Community-based peer support significantly improves metabolic control in people with Type 2 diabetes in Yaounde, Cameroon. Diabetic medicine : a journal of the British Diabetic Association. 2015;32(7):886-9.
18. Baumann LC, Frederick N, Betty N, Jospehine E, Agatha N. A demonstration of peer support for Ugandan adults with type 2 diabetes. International journal of behavioral medicine. 2015;22(3):374-83.
19. Prost A, Colbourn T, Seward N, Houweling TAJ, Fottrell E, Lewycka S, et al. Women's groups practising participatory learning and action to improve maternal and newborn health in low-resource settings: A systematic review and meta-analysis. The Lancet. 2013;381(9879):1736-46.
20. Prost A, Colbourn T, Seward N, Azad K, Coomarasamy A, Copas A, et al. Women's groups practising participatory learning and action to improve maternal and newborn health in low-resource settings: a systematic review and meta-analysis. The Lancet. 2013;381(9879):1736-46.
21. Ahmed SM, Alam BB, Anwar I, Begum T, Huque R, Khan JAM, et al. Bangladesh Health System Review. Health Systems in Transition. 2015;5(3).
22. Michie S, van Stralen MM, West R. The behaviour change wheel: a new method for characterising and designing behaviour change interventions. Implementation science : IS. 2011;6:42.
23. Michie SJ, M.; Abraham, C.; Lawton, R.; Parker, D.; Walker, A., on behalf of the 'Psychological Theory' Group. Making psychological theory useful for implementing evidence based practice: a consensus approach. Qual Saf Health Care. 2005(14):26-33.
24. Michie S, Richardson M, Johnston M, Abraham C, Francis J, Hardeman W, et al. The behavior change technique taxonomy (v1) of 93 hierarchically clustered techniques: building an international consensus for the reporting of behavior change interventions. Ann Behav Med. 2013;46(1):81-95.
25. Fottrell E, Azad K, Kuddus A, Younes L, Shaha S, Nahar T, et al. The effect of increased coverage of participatory women's groups on neonatal mortality in Bangladesh: a cluster randomized trial. JAMA Pediatrics. 2013;167(9):816-25.
26. Klose T. The contingent valuation method in health care. Health Policy. 1999;47(2):97-123.
27. O'Brien B, Gafni A. When do the "dollars" make sense? Toward a conceptual framework for contingent valuation studies in health care. Med Decis Making. 1996;16(3):288-99.
28. Diener A, O'Brien B, Gafni A. Health care contingent valuation studies: a review and classification of the literature. Health Econ. 1998;7(4):313-26.
29. Hansen KS, Pedrazzoli D, Mbonye A, Clarke S, Cundill B, Magnussen P, et al. Willingness-to-pay for a rapid malaria diagnostic test and artemisinin-based combination therapy from private drug shops in Mukono District, Uganda. Health Policy Plan. 2013;28(2):185-96.

For peer review only - http://bmjopen.bmj.com/site/about/guidelines.xhtml

30. Shariful Islam SM, Lechner A, Ferrari U, Seissler J, Holle R, Niessen LW. Mobile phone use and willingness to pay for SMS for diabetes in Bangladesh. J Public Health (Oxf). 2016;38(1):163-9.
31. Russell S, Fox-Rushby J, Arhin D. Willingness and ability to pay for health care: a selection of methods and issues. Health Policy Plan. 1995;10(1):94-101.
32. Byford S, Raftery J. Perspectives in economic evaluation. BMJ. 1998;316(7143):1529-30.
33. Jonsson B. Ten arguments for a societal perspective in the economic evaluation of medical innovations. Eur J Health Econ. 2009;10(4):357-9.
34. Fiedler JL, Villalobos CA, De Mattos AC. An activity-based cost analysis of the Honduras community-based, integrated child care (AIN-C) programme. Health Policy Plan. 2008;23(6):408-27.
35. Johns B, Baltussen R, Hutubessy R. Programme costs in the economic evaluation of health interventions. Cost Eff Resour Alloc. 2003;1(1):1.
36. Batura N, Pulkki-Brannstrom AM, Agrawal P, Bagra A, Haghparast-Bidgoli H, Bozzani F, et al. Collecting and analysing cost data for complex public health trials: reflections on practice. Global health action. 2014;7:23257.
37. Hutton G, Baltussen R. Cost valuation in resource-poor settings. Health Policy Plan. 2005;20(4):252-9.
38. Walker D. Cost and cost-effectiveness guidelines: which ones to use? Health Policy Plan. 2001;16(1):113-21.
39. Conteh L, Walker D. Cost and unit cost calculations using step-down accounting. Health Policy Plan. 2004;19(2):127-35.
40. Gray AM, Clarke PM, Wolstenholme JL, Wordsworth S. Applied Methods of Cost-effectiveness Analysis in Healthcare (Handbooks in Health Economic Evaluation). Oxford Oxford University Press; 2011.
41. Drummond MF, Sculpher MJ, Claxton K, Stoddart GL, Torrance GW. Methods for the economic evaluation of health care programmes: Oxford university press; 2015.
42. NICE. How NICE Measures Value for Money in Relation to Public Health Interventions. London: NICE (http://www.nice.org.uk) 2013.
43. Tan-Torres Edejer T BR, Adam T, Hutubessy R, Acharya A, Evans DB, Murray CJ. Making choices in health: WHO guide to cost-effectiveness analysis. Geneva: World Health Organization; 2003.
44. Claxton K, Revill P, Sculpher M, Wilkinson T, Cairns J, Briggs A. The Gates Reference Case for Economic Evaluation. The Bill and Melinda Gates Foundation; 2014.
45. Husereau D, Drummond M, Petrou S, Carswell C, Moher D, Greenberg D, et al. Consolidated Health Economic Evaluation Reporting Standards (CHEERS)--explanation and elaboration: a report of the ISPOR Health Economic Evaluation Publication Guidelines Good Reporting Practices Task Force. Value Health. 2013;16(2):231-50.
46. Woods B, Revill P, Sculpher M, Claxton K. Country-Level Cost-Effectiveness Thresholds: Initial Estimates and the Need for Further Research. Value Health. 2016;19(8):929-35.
47. Verguet S, Kim JJ, Jamison DT. Extended Cost-Effectiveness Analysis for Health Policy Assessment: A Tutorial. Pharmacoeconomics. 2016;34(9):913-23.

For peer review only

For peer review only

For peer review only - http://bmjopen.bmj.com/site/about/guidelines.xhtml

Table 1: Overview of resource use and costs measures included in the economic evaluation of the D-Magic interventions.

Perspective/cost category		Type of costs	Description	Sources	Sample size
Provider	Program/implementing agency	Direct	Costs of implementing mHealth, community mobilisation and HSS interventions	(1) Project accounts of the implementing agencies (2) Interviews with the project staff	N/A
		Indirect	The opportunity cost of volunteer experts attended the mHealth design meetings, donated items, etc.	(1) Project records on numbers of meeting, attendants etc. (2) Published reports on local wage information based on skill category (3) Field offices' inventory information	(1) All meetings held and number of people attended in the meetings will be recorded (2) The list of all equipment in the field offices
	Public healthcare providers	Direct	Changes in utilisation of T2DM and NCD related services at the public health facilities in the study area	(1) Detailed audit and costing study of the health facilities (2) Baseline and end-line cross-sectional surveys (for information on changes in costs of health seeking)	(1) Random sample of health facilities at different levels in both intervention and control areas (2) All participants in the study
		Indirect	The opportunity cost of the time spent by the health care providers attending HSS meetings	(1) Project records on numbers of meeting, attendants etc. (2) Published reports on local wage information based on skill category	All meetings held and number of people attended in the meetings will be recorded
Participants/households		Direct	Household expenditure on food and non-food	Household consumption expenditure survey	A random sample of 300 households in the study area
			Costs of health seeking for the participants and their households	Baseline and end-line cross-sectional surveys	All participants in the study
		Indirect	Opportunity cost of participation in the groups	Group participants survey	A random sample of 312 group participants (both male and female)

* HSS: health system strengthening, T2DM: type 2 Diabetes mellitus, NCD: non-communicable diseases

For peer review only - http://bmjopen.bmj.com/site/about/guidelines.xhtml

For peer review only

For peer review only - http://bmjopen.bmj.com/site/about/guidelines.xhtml

CHEERS Tool

Section/item		Item no.	Recommendation	Response
Title and abstract	Title	1	Identify the study as an economic evaluation, or use more specific terms such as ''cost-effectiveness analysis'' and describe the interventions compared.	Yes
	Abstract	2	Provide a structured summary of objectives, perspective, setting, methods (including study design and inputs), results (including base-case and uncertainty analyses), and conclusions.	Yes
Introduction	Background and objectives	3a	Provide an explicit statement of the broader context for the study.	Yes
		3b	Present the study question and its relevance for health policy or practice decisions.	Yes
Methods	Target population and subgroups	4	Describe characteristics of the base-case population and subgroups analyzed including why they were chosen.	Yes
	Setting and location	5	State relevant aspects of the system(s) in which the decision(s) need(s) to be made.	Yes
	Study perspective	6	Describe the perspective of the study and relate this to the costs being evaluated.	Yes
	Comparators	7	Describe the interventions or strategies being compared and state why they were chosen.	Yes
	Time horizon	8	State the time horizon(s) over which costs and consequences are being evaluated and say why appropriate.	Yes
	Discount rate	9	Report the choice of discount rate(s) used for costs and outcomes and say why appropriate.	Yes
	Choice of health outcomes	10	Describe what outcomes were used as the measure(s) of benefit in the evaluation and their relevance for the type of analysis performed.	Yes
	Measurement of effectiveness	11 a)	Single study–based estimates: Describe fully the design features of the single effectiveness study and why the single study was a sufficient source of clinical effectiveness data.	Yes
		11 b)	Synthesis-based estimates: Describe fully the methods used for the identification of included studies and synthesis of clinical effectiveness data.	n/a
	Measurement and valuation of preference based outcomes	12	If applicable, describe the population and methods used to elicit preferences for outcomes.	n/a
	Estimating resources and costs	13 a)	Single study–based economic evaluation: Describe approaches used to estimate resource use associated with the alternative interventions. Describe primary or secondary research methods for valuing each resource item in terms of its unit cost. Describe any adjustments made to approximate to opportunity costs.	Yes
		13 b	Model-based economic evaluation: Describe approaches and data sources used to estimate resource use associated with model health states. Describe primary or secondary research methods for valuing each resource item in terms of its unit cost. Describe any adjustments made to approximate to opportunity costs.	n/a

	Currency, price date, and conversion	14	Report the dates of the estimated resource quantities and unit costs. Describe methods for adjusting estimated unit costs to the year of reported costs if necessary. Describe methods for converting costs into a common currency base and the exchange rate.	Yes
	Choice of model	15	Describe and give reasons for the specific type of decision-analytic model used. Providing a figure to show model structure is strongly recommended.	n/a
	Assumptions	16	Describe all structural or other assumptions underpinning the decision-analytic model.	n/a
	Analytical methods	17	Describe all analytic methods supporting the evaluation. This could include methods for dealing with skewed, missing, or censored data; extrapolation methods; methods for pooling data; approaches to validate or make adjustments (e.g., half-cycle corrections) to a model; and methods for handling population heterogeneity and uncertainty.	Yes
Results	Study parameters	18	Report the values, ranges, references, and if used, probability distributions for all parameters. Report reasons or sources for distributions used to represent uncertainty where appropriate. Providing a table to show the input values is strongly recommended.	Not possible in the protocol although the process is described
	Incremental costs and outcomes	19	For each intervention, report mean values for the main categories of estimated costs and outcomes of interest, as well as mean differences between the comparator groups. If applicable, report incremental cost-effectiveness ratios.	Not possible in the protocol although the process is described
	Characterising uncertainty	20 a	Single study–based economic evaluation: Describe the effects of sampling uncertainty for estimated incremental cost, incremental effectiveness, and incremental cost-effectiveness, together with the impact of methodological assumptions (such as discount rate, study perspective).	Not possible in the protocol although the process is described
		20 b	Model-based economic evaluation: Describe the effects on the results of uncertainty for all input parameters, and uncertainty related to the structure of the model and assumptions.	n/a
	Characterising heterogeneity	21	If applicable, report differences in costs, outcomes, or cost-effectiveness that can be explained by variations between subgroups of patients with different baseline characteristics or other observed variability in effects that are not reducible by more information.	Not possible in the protocol although the process is described
Discussion	Study findings, limitations, generalisability, and current knowledge	22	Summarize key study findings and describe how they support the conclusions reached. Discuss limitations and the generalizability of the findings and how the findings fit with current knowledge	Not possible in the protocol although the process is described
Other	Source of funding	23	Describe how the study was funded and the role of the funder in the identification, design, conduct, and reporting of the analysis. Describe other nonmonetary sources of support.	Yes
	Conflicts of interest	24	Describe any potential for conflict of interest among study contributors in accordance with journal policy. In the absence of a journal policy, we recommend authors comply with International Committee of Medical Journal Editors' recommendations.	Yes

BMJ Open

Protocol of economic evaluation and equity impact of m-Health and community groups for prevention and control of diabetes in rural Bangladesh: a three-arm cluster randomised controlled trial.

Journal:	*BMJ Open*
Manuscript ID	bmjopen-2018-022035.R1
Article Type:	Protocol
Date Submitted by the Author:	16-May-2018
Complete List of Authors:	Haghparast-Bidgoli, Hassan; University College London, Institute for Global Health<br>Shaha, Sanjit ; Diabetic Association of Bangladesh<br>Kuddus, Abdul; Diabetic Association of Bangladesh, Perinatal Care Project<br>Chowdhury, Md.Alimul Reza ; Diabetic Association of Bangladesh<br>Jennings, Hannah ; University College London, Institute for Global Health<br>Ahmed, Naveed; Diabetic Association of Bangladesh<br>Morrison, Joanna; University College London, Institute for Global Health<br>Akter, Kohenour; Diabetic Association of Bangladesh<br>Nahar, Badrun ; Diabetic Association of Bangladesh<br>Nahar, Tasmin ; Diabetic Association of Bangladesh<br>King, C.; UCL, Institute for Global Health<br>Skordis-Worrall, Jolene; University College London Medical School,<br>Batura, Neha; University College London, Institute for Global Health<br>Khan, Jahangir; Liverpool School of Tropical Medicine, Health Economics<br>Mansaray, Anthony ; ICAP Sierra Leone<br>Hunter, Rachael; University College London, Research Dept of Primary Care and Population Health<br>Azad Khan, A.K. ; Diabetic Association of Bangladesh<br>Costello, Anthony ; University College London, Institute for Global Health; World Health Organisation<br>Azad, Kishwar; Diabetic Association of Bangladesh,<br>Fottrell, Edward; University College London, Institute for Global Health
**Primary Subject Heading**:	Health economics
Secondary Subject Heading:	Diabetes and endocrinology, Global health, Health policy, Health services research, Public health
Keywords:	HEALTH ECONOMICS, DIABETES & ENDOCRINOLOGY, Health policy < HEALTH SERVICES ADMINISTRATION & MANAGEMENT

SCHOLARONE™
Manuscripts

For peer review only

Protocol of economic evaluation and equity impact of m-Health and community groups for prevention and control of diabetes in rural Bangladesh: a three-arm cluster randomised controlled trial.

Hassan Haghparast-Bidgoli[1*], Sanjit Kumar Shaha[2], Abdul Kuddus[2], Md.Alimul Reza Chowdhury[2], Hannah Jennings[1], Naveed Ahmed[2], Joanna Morrison[1], Kohenour Akter[2], Badrun Nahar[2], Tasmin Nahar[2], Carina King[1], Jolene Skordis[1], Neha Batura[1], Jahangir A. M. Khan[4], Anthony Mansaray[5], Rachael Hunter[6], A.K. Azad Khan[2], Anthony Costello[1, 3], Kishwar Azad[2], Edward Fottrell[1]

1 University College London, Institute for Global Health, London, United Kingdom
2 Diabetic Association of Bangladesh, Dhaka, Bangladesh
3 World Health Organisation, Geneva, Switzerland
4 Liverpool School of Tropical Medicine, Liverpool, United Kingdom
5 ICAP Sierra Leone, Sierra Leone.
6 University College London, Institute of Epidemiology & Health, London, United Kingdom

*Corresponding author
Hassan Haghparast-Bidgoli
Address: University College London, Institute for Global Health, 30 Guilford Street, London WC1N 1EH, UK
Email: h.haghparast-bidgoli@ucl.ac.uk
Tel: +44 (0) 20 7905 2779

Word count: 4437

For peer review only

For peer review only - http://bmjopen.bmj.com/site/about/guidelines.xhtml

Abstract

Introduction: Type 2 Diabetes mellitus (T2DM) is one of the leading causes of death and disability worldwide, generating substantial economic burden for people with diabetes and their families, and to health systems and national economies. Bangladesh has one of the largest numbers of adults with diabetes in the South Asian region. This paper describes the planned economic evaluation of a three-arm cluster randomised control trial of mHealth and community mobilisation interventions to prevent and control T2DM and non-communicable diseases' risk factors in rural Bangladesh (D-Magic trial).

Methods and Analysis: The economic evaluation will be conducted as a within-trial analysis to evaluate the incremental costs and health outcomes of mHealth and community mobilisation interventions compared to the status quo. The analyses will be conducted from a societal perspective, assessing the economic impact for all parties affected by the interventions, including implementing agencies (program costs), health care providers, and participants and their households. Incremental cost-effectiveness ratios (ICERs) will be calculated in terms of cost per case of intermediate hyperglycaemia and T2DM prevented and cost per case of diabetes prevented among individuals with intermediate hyperglycaemia at baseline, and cost-per mm Hg reduction in systolic blood pressure. In addition to ICERs, the economic evaluation will be presented as a cost-consequence analysis where the incremental costs and all statistically significant outcomes will be listed separately. Robustness of the results will be assessed through sensitivity analyses. In addition, an analysis of equity impact of the interventions will be conducted.

Ethics and Dissemination: The approval to conduct the study was obtained by the University College London Research Ethics Committee (4766/002) and by the Ethical Review Committee of the Diabetic Association of Bangladesh (BADAS-ERC/EC/t5100246). The findings of this study will be disseminated through different means within academia and the wider policy sphere.

Trial registration: ISRCTN41083256. Registered 30/03/2016

For peer review only - http://bmjopen.bmj.com/site/about/guidelines.xhtml

Strengths and limitations of this study

- This protocol paper reports planned data collection and analyses alongside a complex public health trial to ensure transparency.
- This protocol can assist in designing mHealth and community mobilisation through Participatory Learning and Action Approach interventions for the prevention of diabetes and mitigation of NCDs risk factors.
- The protocol, and planned analysis and reporting follow recommended guidelines to design and report economic evaluations.
- An equity impact analysis and contingent valuation study will be conducted alongside the study.
- The study is powered to assess the cost-effectiveness of each intervention against control only. The study is not powered to test the differences between the mHealth and community mobilisation interventions. Therefore, it will be possible to estimate incremental cost and effect of each intervention compared to the control only.

For peer review only

For peer review only - http://bmjopen.bmj.com/site/about/guidelines.xhtml

BACKGROUND

Burden of diabetes mellitus

Diabetes mellitus, mainly type 2, is a one of the leading causes of death and disability worldwide. It is estimated that around 415 million people worldwide, or 9% of adults aged 20-79 have diabetes, with about 75% of people living with diabetes residing in low- and middle-income countries (LMICs) (1). It is predicted that by 2040, one in 10 adults will have diabetes, unless preventive efforts are undertaken (1). Diabetes, if not managed properly, can lead to several complications, such as heart attack, kidney failure, leg amputation, vision loss and several other long-term consequences, that impact significantly on quality of life and cause premature death (1, 2). Diabetes and its complications create a substantial economic burden for people with diabetes and their families, and for health systems and national economies through direct medical costs and productivity loss (2). It is reported that in many countries between 5% and 20% of total health expenditure are spent on diabetes (1). This is in addition to the large financial burden on individuals and their families due to the cost of seeking care.

Bangladesh has the second largest number of adults with diabetes in the South Asian region, with around 7 million adults aged 20-79 years with diabetes (1). The prevalence of diabetes among adults (20-79 years old) in Bangladesh is estimated to be around 8.5 % (1, 2), with a three to four-fold increase since the 1990s (3, 4). It is predicted that prevalence of diabetes in Bangladesh will reach 23.6% in men and 33.5% in women by 2030, unless preventive efforts are undertaken (3). The increasingly high incidence of diabetes in Bangladesh has had a significant economic burden, in particular for people with diabetes, their families and the country's health care system. It is estimated that the annual health expenditure for diabetes is around US$218 million, where most of the costs born by the families (5).

Underlying the increasing prevalence of diabetes, globally and in Bangladesh, are complex genetic, environmental and lifestyle factors, including changes in dietary habits and increases in risk factors like smoking and physical inactivity (2). Effective interventions are available to prevent type 2 Diabetes mellitus (T2DM), and to prevent the complications and premature death from diabetes (2). The evidence from LMICs shows that lifestyle and other non-pharmacological interventions can prevent and delay the onset of T2DM and its complications (6). However, there is a lack of cost effective programs designed specifically for Bangladeshi populations, taking into account their contextual needs and resources.

The Bangladesh D-Magic Trial

The Bangladesh D-magic is a three-arm cluster randomised control trial (cRCT) of mHealth and community mobilisation interventions, conducted in four rural upazilas in Faridpur district, Bangladesh. The trial aims to assess the effectiveness and cost-effectiveness of the interventions in the prevention and control of T2DM and NCD risk factors in rural Bangladesh. In the D-magic trial, the clusters (96 villages) were

For peer review only - http://bmjopen.bmj.com/site/about/guidelines.xhtml

randomised to receive either the mHealth intervention, the community mobilisation intervention, or be in the control arm (32 in each). In the mHealth intervention, individuals receive voice messages about prevention and control of NCD risk factors and T2DM on their mobile phone. In the community mobilisation intervention, a trained facilitator initiates a series of diabetes and NCD risk-factor focused monthly group meetings for men and women, working through a participatory learning and action (PLA) cycle by which group members themselves identify, prioritise and tackle problems associated with T2DM and its risk factors. In addition, all study areas (both intervention and control) clusters receive health system strengthening activities, which include the training/refresher training of health care workers working in the community and health facilities, in the prevention, diagnosis and treatment of T2DM, as well as the development of essential equipment inventories.

Details of the D-Magic trial are described in detail elsewhere (7). This protocol paper aims to fully describe the methodology for the economic evaluation of the trial.

Economic evaluations of community and mHealth interventions for prevention and control of diabetes

There is evidence that mHealth programs can have a positive impact on behaviour change and prevention and control of diabetes and NCDs in high-risk populations (8-11). However, there is little information on the cost and cost-effectiveness of mHealth interventions for the prevention and control of NCDs (12-14). Two recent systematic reviews of the economic evidence of mHealth (13), and mHealth for diabetes prevention and control (14), have shown that there are a handful of NCD and diabetes interventions that have reported cost and cost effectiveness evidence. Nearly all of these studies have been conducted in high-income settings. The majority of these studies report that mHealth interventions are cost-effective or cost saving, though the quality of reported evidence was not satisfactory in some of cases (13, 14).

Similarly, although there is some evidence on effectiveness of community-based interventions in the management of T2DM in low-income settings (15-19), there is little evidence on how cost effective these interventions might be (14). A recent review (14) has identified 10 community-based interventions on preventing and controlling diabetes. These interventions, which are largely implemented in high-income settings, have reported that community-based interventions are cost-effective or cost-saving approaches in the management of T2DM (14).

The current study will be the first to assess the cost-effectiveness of community mobilisation through PLA in the prevention of T2DM. This approach has previously been shown to be highly cost-effective in improving maternal and newborn health (20, 21). This study will also contribute to the evidence on cost-effectiveness of mHealth interventions for preventing T2DM in LMIC settings.

Aim and objectives

For peer review only - http://bmjopen.bmj.com/site/about/guidelines.xhtml

The D-Magic economic evaluation aims to measure the cost-effectiveness of mHealth and participatory community group interventions to prevent and to control T2DM and NCD risk factors in rural Bangladesh from a societal perspective.

The specific objectives of the D-Magic economic evaluation are:

1. To estimate the costs of setting up and implementing the mHealth and participatory community group interventions as well as health system strengthening (HSS) activities;
2. To calculate the costs to the healthcare system, of increased care-seeking (i.e. diagnosis and treatment) for T2DM and other NCDs, as a result of the D-Magic interventions;
3. To measure costs associated to the intervention participants and their households of changes in diabetes or other NCD-related diagnosis and management care-seeking costs as well as any costs associate with changes in diet and other life-style behaviours, as a result of the D-Magic interventions;
4. To present the incremental costs and outcomes of the interventions as a cost-consequence analysis;
5. To calculate the incremental cost-effectiveness of the mHealth and community mobilisation interventions combined with HSS activities, as compared to HSS activities alone, where all new HSS activities are delivered in addition to the existing government programmes.

In addition to the above-mentioned objectives, the equity impact of the mHealth and community mobilisation interventions will be assessed.

METHODS

Study setting and population

The study setting for the D-Magic trial is Faridpur district, located south of Dhaka. The district has a population of over 1.7 million people in a high-density area of just over 2000 square kilometres. It is primarily an agricultural economy, with the main crops being jute and rice. Like in the rest of Bangladesh, healthcare is provided at three levels: primary care is provided at Community Clinics and at Union Health and Family Welfare Centres; secondary level care providers (both in- and out-patient services) are sub-district (Upazilla) health complexes and hospitals; and tertiary care is provided at district hospitals and medical college hospitals (22). Private and informal service providers are also present in the area, with the informal sector being the main provider in rural areas (22), Faridpur district included. Inadequate and inequitable access to services, shortages of skilled health care providers, short supplies of medicines and poor quality and low responsiveness of services are the main challenges faced by the Bangladesh healthcare system (22), and remains a challenge in Faridpur district too.

The study population for the D-Magic study are men and non-pregnant women who are aged 30 years or more and permanent residents (i.e. lived there for more than 6 month) of 96 villages (clusters) in four rural upazillas – Nagarkanda, Boalmari, Saltha and Madhukhali – in Faridpur district.

For peer review only - http://bmjopen.bmj.com/site/about/guidelines.xhtml

Trial design

The D-Magic trial is a three-arm cRCT which is implemented in four rural upazilas in Faridpur district, Bangladesh. In D-Magic trial, 96 clusters (villages) were randomised to receive either the mHealth intervention, the community mobilisation intervention, or be in the control arm. All three arms receive a number of health system strengthening activities. The interventions implementation was started in July 2016 and will be completed by end of December 2017 and all data collection will be ongoing until June 2018.

Detailed information on the randomisation and participants recruitment process, trial time-line, and a full description of the interventions is presented elsewhere (7). A brief description of the mHealth, community mobilisation and health system strengthening interventions are presented in the following sections.

mHealth intervention

The mHealth intervention involves free of charge voice messages about the prevention and control of T2DM and NCD risk factors sent twice-weekly to the individual's mobile phone during a 14-month period. The intervention and the messages' content were developed based on findings from baseline formative research in the study area, and application of the behaviour change theories the COM-B (Capability, Opportunity, Motivation, Behaviour) model for understanding behaviour (23) and the Theoretical Domains Framework (TDF) to encourage change (24, 25). The mHealth intervention is available to all individuals who have access to a mobile phone and registered to receive the messages by providing their mobile number to the intervention community recruiters.

Community Mobilisation intervention

The community mobilisation intervention involves initiation and facilitation of separate male and female participatory groups, with approximately 20 members in each. The intervention is an adaptation of a participatory women's groups intervention implemented in South Asia (including Bangladesh) and Sub-Saharan African settings and shown to be effective and cost effective at reducing neonatal mortality (21, 26). The groups progress through a series of 18 monthly meetings following the four phases of PLA. During phase 1, participants identify and prioritise factors that affect their health, particularly those increasing their risk of developing or failing to manage T2DM; in phase 2, the participants and their community come up with feasible strategies that can be implemented to address the problems identified in phase 1; during phase 3, they implement these strategies; in phase 4 they evaluate the strategies they have implemented. The groups are run by salaried facilitators, who are recruited from the study areas and have a minimum of higher secondary school education. The facilitators undertook one week's training on group facilitation and basic health messages related to NCD prevention and control, in particular T2DM. They also have received refresher training during the course of the interventions.

Health Systems Strengthening (HSS)

For peer review only - http://bmjopen.bmj.com/site/about/guidelines.xhtml

A series of health system strengthening activities are carried out in all study areas (both intervention and control clusters). These activities, which are tailored according to the mapping of health care providers in the project area in the formative phase of the project, included the training of mainly informal health care workers in the community and distributing educational materials among formal and informal providers in prevention, diagnosis and treatment of T2DM, as well as the development of essential equipment inventories.

Measurement of health outcomes/effectiveness

The D-Magic trial will test the effect of the community mobilisation intervention and mHealth intervention relative to the control and does not directly compare the effects of each intervention relative to the other. As this is a cluster-randomised trial, the outcomes will be measured among individuals (permanent residents) who live in the intervention clusters, irrespective of whether they took part in groups or received mHealth messages. Analysis of the outcomes will be by intention to treat at the individual and cluster level as appropriate. Moreover, participants with missing data on the primary outcomes will be excluded from primary outcome analysis (7).

Primary outcome

The D-Magic trial has two primary outcomes: (1) combined prevalence of intermediate hyperglycaemia (i.e. impaired fasting glucose or impaired glucose tolerance) and T2DM, and (2) cumulative two-year incidence of T2DM among individuals identified with intermediate hyperglycaemia at the start of the trial (7). Using the prevalence data, the number of intermediate hyperglycaemia and diabetes mellitus cases prevented, and the number of diabetes cases prevented among individuals with intermediate hyperglycaemia at baseline will be calculated as the difference between the expected and the actual number of cases using the adjusted odds ratio relative to the control population.

Secondary outcomes

The trial has a number of secondary outcomes including diastolic and systolic blood pressure, prevalence of hypertension, body mass index, prevalence of overweight and obesity, prevalence of abdominal obesity, health-related quality of life, and psychological distress among self-reported diabetics (7).

Comparison will be made between the interventions (mHealth and community mobilisation) and control (HSS activities only) to estimate incremental cost-effectiveness ratios (ICERs) for the primary outcomes; in terms of cost per case of intermediate hyperglycaemia and diabetes mellitus prevented and cost per case of diabetes prevented among individuals with intermediate hyperglycaemia at baseline, and for some of the secondary outcomes such as cost- per mm Hg reduction in systolic BP. ICER will be conducted if a significant impact on the outcomes is observed. Moreover, all costs and (statistically significant) outcomes, both primary and secondary, will be presented separately in a cost-consequence analysis.

For peer review only - http://bmjopen.bmj.com/site/about/guidelines.xhtml

Willingness to pay/Contingent Valuation study

We will conduct a willingness to pay (WTP) study in order to elicit maximum monthly amount of money each participant or household would be willing to pay if an m-Health service on diabetes and NCD risk factors' prevention and management (i.e. weekly voice messages) was available.

WTP studies are widely used in order to elicit monetary value of a service or good not available in the market (27-30). In the Bangladesh context, Islam et al (2015) in a recent study estimated WTP of T2DM patients for receiving messages for increasing adherence to treatment (31). Similar to Islam et al (2015), we will use an open-ended contingent valuation (CV) method (27, 30, 32), asking participants through an open-ended question how much they would be willing to pay monthly to receive voice messages related to diabetes and NCD risk factors' prevention and management, if such a service was available. Open-ended CV is a more flexible approach and avoids starting point bias or range bias introduced by other methods such bidding game and payment cards (27). The theoretical framework defined by O'Brien and Gafni (28) for CV studies will be used to design the study.

WTP questions will be asked from all the participants at the end-line impact evaluation survey. WTP values will be compared across different groups, for example, based on exposure to the interventions (i.e. among those exposed to mHealth, or exposed to participatory groups, and participants who have not been exposed to either of these interventions) or health condition (people diagnosed with diabetes or other NCDs and others). In addition, for each participant, detailed individual and household level socio-economic characteristics will be collected to examine the extent to which WTP values will vary by socio-economic status of participants.

Equity impact of the D-Magic interventions

In addition to measuring efficiency/cost-effectiveness of the D-Magic interventions, an analysis of equity impact of the interventions will be conducted to assese whether impacts/gains from the intervention are equitably shared among the target population. This will be done through sub-group analyses of the primary and secondary outcomes based on the socio-economic status of the target population.

Identification, measurement and valuation of resource use

The cost-effectiveness and cost-consequence of the D-Magic interventions will be measured from a societal perspective (33, 34); measuring the economic impact for all parties affected by the interventions, including implementing agency (or program costs), public healthcare providers (at both local and national levels) and users, who are the intervention participants and their households. The following sections provide a detailed description of the proposed methods for measuring and valuing programme costs, healthcare provider costs and participants/household costs. Programme costs include those incurred by the implementing agency or program provider i.e. Diabetic Association of Bangladesh (BADAS). The healthcare provider costs are those incurred by the government health facilities in the study area including community

For peer review only - http://bmjopen.bmj.com/site/about/guidelines.xhtml

clinics, Health & Family Welfare centres and Upazila Health Complex/hospitals. The household or user costs include those incurred by participants and their households. Table 1 provides an overview of the financial and economic costs to be employed in the economic evaluation of the D-Magic interventions.

Programme-related costs

Direct and indirect costs of designing and implementing mHealth, community mobilisation and health system strengthening interventions will be estimated using a combination of activity-based costing (35) and ingredients approach (36).

Financial or expenditure data

Programme costs are mainly financial or accounting costs, which are collected prospectively from the project accounts or expenditure records of the implementing partner and entered (generally, on an annual basis) to an MS Excel data capture tool. The tool contains different sections/worksheets that will allow the categorization of costs into line items (i.e. staff, materials, capital, and joint costs), start up and implementation costs of the interventions, and costs associated with the different programme components, i.e. m-Health, community mobilization, HSS, and monitoring and evaluation. Key informant interviews with project leads and monthly/quarterly staff time sheets will be used to allocate joint costs between the programme components. The summary worksheets in the cost data capture tool present the costs by programme component (e.g. mHealth, community mobilisation and HSS), summarise the total cost data per intervention, allows effect data to be entered and estimates the cost-effectiveness results.

Donated items and opportunity costs

Some items, such as donated items and volunteer time, are not captured in the accounting system and need to be converted to economic costs using their market value and then entered into the data capture tool (37-39). Potential donated items are equipment donated by the implementing agency (i.e. purchased by previous projects and used in the D-Magic project). The donated items will be identified through key informant interviews with the project leads.

The majority of volunteer time is related to designing messages for the mHealth intervention, where several meetings were held with experts who were volunteers. Detailed information regarding these meetings, including the number of meetings, their duration, and the participants is being documented by the project. The opportunity cost of the time invested by the experts will be measured as a proportion of their salary or a salary equivalent using published national/local wage rate reports.

Public healthcare providers costs

The D-Magic project is likely (at least in the short term) to increase seeking care for diabetes, other NCDs and NCD risk factors such as hypertension, particularly demand for services such as testing and treatment for hypertension, diabetes and pre-diabetes, or seeking advice or treatment for weight control. In addition, there is a time

For peer review only - http://bmjopen.bmj.com/site/about/guidelines.xhtml

(opportunity) cost of direct involvement in the HSS activities for the healthcare providers (Table 1).

Cost of changes in demand for services

The costs to public healthcare providers in the project area due to increased (or any changes in) demand for their services will be estimated. A mapping of the health care providers in the study area has been completed and 20 functional governmental health care facilities, at different levels, have been identified. These facilities included 14 Community Clinics, 3 Health & Family Welfare centres and 3 Upazila Health Complex/Hospitals. A sample of 11 health facilities including 8 community clinics and 2 Health & Family Welfare centres (with equal numbers in control and intervention clusters), as well as one Upazila Health Complex/Hospital (which cover both control and intervention areas) were selected for baseline audits of diabetes and NCD services, estimating their resource utilisations and unit costs. Similar audits and cost data collection will be conducted for the same facilities post-intervention in order to assess the changes in NCD service utilisation attributable to the D-Magic interventions. This data will be complemented by health seeking behaviour information collected from the study participants in control and interventions clusters at the D-Magic end-line impact evaluation survey. Differences in service utilisation between intervention and control areas will be attributed to the D-Magic interventions.

A simple audit and cost-capture tool was developed for facility data collection and piloted with facilities at different levels. Data from the cost-capture tool will be complemented by the existing data from the facility reports, and published data. Costs of services provided by the facilities will be estimated using a step-down approach (40).

Any change in demand for services provided in the facilities other than those mentioned above and the services not covered by them, in intervention areas compared to the control, will be identified during the trial's routine monitoring and end-line impact evaluation survey. Any cost of that change in demand will be calculated using published data on the unit costs of those services.

Opportunity cost of HSS activities

Moreover, as discussed earlier, HSS activities include several training sessions for health care providers in prevention, diagnosis and treatment of T2DM. Information on the number of meetings, their duration and participation is being documented by the project. The opportunity cost of the time spent by the providers will be measured as a proportion of their salary for formal providers or as a salary equivalent, for informal providers.

Participants and their household costs

D-Magic interventions may influence participants and their households' costs in a number of ways. These include changes in health seeking behaviour, and changes in household lifestyles that might affect food and non-food consumption patterns and

For peer review only - http://bmjopen.bmj.com/site/about/guidelines.xhtml

spending as well as time spent engaging in physical activities. It also includes the time (opportunity) cost of participation in the PLA group meetings and participating in the actions taken by the groups (Table 1).

Health care seeking costs

D-Magic may increase the participants and their households' seeking advice and care from both formal and informal providers for testing and treatment for hypertension, diabetes, pre-diabetes and other NCDs. Information on costs of care seeking are collected from all the participants recruited in the project, at the baseline and end-line evaluation surveys. This information will be complemented by the data collected from the household consumption expenditure survey. The difference in spending for the participants and their households will be calculated and compared between intervention and control areas.

Changes in household food and non-food expenditure

Changes in food and non-food expenditure will be captured in a comprehensive household consumption and expenditure survey. The survey will be conducted on a random sub-sample of 300 households (100 per trial arm) at the end of intervention period. The changes in the expenditure will be compared between interventions and control areas.

Opportunity cost of participation in the interventions

Participating in the PLA group meetings incur some costs to the participants and their family. These costs include the direct costs (e.g. cost of getting to the group) and time cost of group participation (e.g. travel time and time spent in the group) and participating in the actions taken by the group, or changing health and lifestyle behaviours. Information on the potential direct and time costs will be collected through a sub-sample survey of 312 group participants (both male and female), randomly selected. Sample size for the survey was primarily calculated to give sufficiently accurate estimate of group participants' characteristics.

Cost-effectiveness and cost-consequence analyses

Economic evaluation will be conducted as a within-trial analysis using the intention-to-treat results, and will be presented in terms of ICERs, calculated as the difference in total costs of mHealth and community mobilisation interventions (plus HSS activities) versus HSS activities only (or control), divided by the difference in mean effects of each interventions versus control (41) (42). As mentioned previously, ICERs will be evaluated in terms of cost per case of intermediate hyperglycaemia and T2DM prevented and cost per case of T2DM prevented among individuals with intermediate hyperglycaemia at baseline, and for some of the secondary outcomes such as cost- per mm Hg reduction in systolic BP. In addition to ICERs, the economic evaluation will be presented as a cost-consequence analysis where the incremental costs and all statistically significant outcomes will be listed separately, allowing policymakers to compare the costs and all impacts/gains of the D-Magic

For peer review only

For peer review only - http://bmjopen.bmj.com/site/about/guidelines.xhtml

interventions. Cost-consequence analysis has been recommended for complex public health interventions, such as D-Magic, that have multiple health and non-health impacts, which are difficult to measure in a common outcome unit (42, 43).

All costs will be presented in 2017 prices in Bangladeshi Taka and International Dollars (INT$). All costs will be adjusted for inflation using the Bangladeshi Consumer Price Index (CPI) and will be converted to 2017 INT$ using the Purchasing Power Parity (PPP) conversion factor for Bangladesh. Moreover, both costs and outcomes will be discounted using a standard discount rate of 3%, as recommended by WHO-CHOICE (44) and the Gates Reference Case for Economic Evaluation (45). The impact of uncertainty in key parameters on the cost-effectiveness results will be assessed through a series of deterministic and probabilistic sensitivity analyses. Reporting of the study design, analytical methods and findings will follow the Consolidated Health Economic Evaluation Reporting Standards (CHEERS) statement (46). The D-Magic interventions will be judged to be cost-effective and affordable (though, indirectly) against the WHO-CHOICE recommendation (44), as well as recently developed cost-effectiveness thresholds (47).

The possibility of conducting an extended cost-effectiveness analysis (48) alongside modeling national scale up of the D-Magic interventions will be explored. Moreover, we will explore the possibility of running a long-term cost-effectiveness analysis using decision analytical modelling based on the relevant outcomes such as systolic blood pressure, body mass index, etc. (if statistically significant) to predict future economic impacts from implementing the D-Magic interventions on the target population.

Strengths and limitations of the study

In order to increase transparency and minimize bias, publication and peer-review of economic evaluation protocols is encouraged. This study reports planned data collection and analyses alongside a complex public health trial. The study will contribute to the scarce cost-effectiveness evidence on mHealth and community mobilisation interventions for preventing diabetes and NCDs.

Furthermore, adopting a cost-consequence analysis approach makes it possible to report all health and non-health impacts of the D-Magic interventions, in addition to ICERs, which can assist policy makers to make informed decisions in designing or implementing similar complex interventions. Incorporating equity impact analysis and contingent valuation are other strengths of this study, which provide useful information for future scale-up of the interventions.

The study design has one limitation. The D-Magic trial is not powered to test the differences between the mHealth and community mobilisation interventions due to the large sample size required and the resources available for the trial. However, the possibility of a direct comparison between the two interventions will be explored.

Patient and public involvement

For peer review only - http://bmjopen.bmj.com/site/about/guidelines.xhtml

Patients and public were not involved in the process of this study. Patients and public will be informed of the study results via peer-reviewed journals, conference and local dissemination meetings.

Contributors

HHB, EF, AK, HJ, JM, CK, JS, NB, JK, RH, AKAK, AC and KA contributed to the study design; HHB, EF, CK contributed to the statistical analyses; SKS, MARC, NA, KA, BN and TN contributed to data acquisition; AM has contributed to the literature review and drafting the paper; HHB was responsible for the initial drafting of this manuscript; all authors contributed to the review of this manuscript and provided comments. All authors read and approved the final manuscript.

Funding

The trial is funded by the Medical Research Council UK (MR/M016501/1) under the Global Alliance for Chronic Diseases (GACD) Diabetes Programme.

Competing interests

The authors declare they have no competing interests.

Ethics approval

The D-Magic trial has been approved by the University College London Research Ethics Committee (4766/002) and by the Ethical Review Committee of the Diabetic Association of Bangladesh (BADAS-ERC/EC/t5100246). The trial has been registered and assigned an International Standard Randomised Controlled Trial Number (ISRCTN41083256). Informed cluster-level consent from village/community leaders, and individual informed consent was obtained from all participants in the trial prior to their participation in the study.

References

1. International Diabetes Federation. Diabetes Atlas: Seventh Edition. Brussels, Belgium: International Diabetes Federation; 2015.
2. World Health Organization. Global report on diabetes. Geneva, Switzerland World Health Organization; 2016.
3. Rahman MM, Akter S, Jung J, Rahman MS, Sultana P. Trend, projection, and appropriate body mass index cut-off point for diabetes and hypertension in Bangladesh. Diabetes research and clinical practice. 2017;126:43-53.
4. Saquib N, Saquib J, Ahmed T, Khanam MA, Cullen MR. Cardiovascular diseases and type 2 diabetes in Bangladesh: a systematic review and meta-analysis of studies between 1995 and 2010. BMC public health. 2012;12:434.
5. Sarker AR, Sultana M. Health and economic burden of diabetes in Bangladesh: Priorities for attention and control. Journal of diabetes. 2017;9(12):1118-9.
6. Rawal LB, Tapp RJ, Williams ED, Chan C, Yasin S, Oldenburg B. Prevention of type 2 diabetes and its complications in developing countries: a review. International journal of behavioral medicine. 2012;19(2):121-33.
7. Fottrell E, Jennings H, Kuddus A, Ahmed N, Morrison J, Akter K, et al. The effect of community groups and mobile phone messages on the prevention and

For peer review only

control of diabetes in rural Bangladesh: study protocol for a three-arm cluster randomised controlled trial. Trials. 2016;17(1):600.
8. Hamine S, Gerth-Guyette E, Faulx D, Green BB, Ginsburg AS. Impact of mHealth chronic disease management on treatment adherence and patient outcomes: a systematic review. Journal of medical Internet research. 2015;17(2):e52.
9. Shaw R, Bosworth H. Short message service (SMS) text messaging as an intervention medium for weight loss: A literature review. Health informatics journal. 2012;18(4):235-50.
10. Shetty AS, Chamukuttan S, Nanditha A, Raj RK, Ramachandran A. Reinforcement of adherence to prescription recommendations in Asian Indian diabetes patients using short message service (SMS)--a pilot study. The Journal of the Association of Physicians of India. 2011;59:711-4.
11. Ramachandran A, Snehalatha C, Ram J, Selvam S, Simon M, Nanditha A, et al. Effectiveness of mobile phone messaging in prevention of type 2 diabetes by lifestyle modification in men in India: a prospective, parallel-group, randomised controlled trial. The lancet Diabetes & endocrinology. 2013;1(3):191-8.
12. Stephani V OD, Quentin W,. Does mHealth contribute to improved care for people with non-communicable diseases in developing countries? A systematic review of randomized controlled trials. Berlin: Universitätsverlag der TU Berlin; 2015.
13. Iribarren SJ, Cato K, Falzon L, Stone PW. What is the economic evidence for mHealth? A systematic review of economic evaluations of mHealth solutions. PloS one. 2017;12(2):e0170581.
14. Mansaray A, Haghparast-Bidgoli H. Economic evidence on mHealth and community mobilisation interventions for the prevention and management of type 2 diabtes: A systematic review. 2017.
15. Balagopal P, Kamalamma N, Patel TG, Misra R. A community-based diabetes prevention and management education program in a rural village in India. Diabetes care. 2008;31(6):1097-104.
16. Balagopal P, Kamalamma N, Patel TG, Misra R. A community-based participatory diabetes prevention and management intervention in rural India using community health workers. The Diabetes educator. 2012;38(6):822-34.
17. Sathish T, Williams ED, Pasricha N, Absetz P, Lorgelly P, Wolfe R, et al. Cluster randomised controlled trial of a peer-led lifestyle intervention program: study protocol for the Kerala diabetes prevention program. BMC public health. 2013;13:1035.
18. Assah FK, Atanga EN, Enoru S, Sobngwi E, Mbanya JC. Community-based peer support significantly improves metabolic control in people with Type 2 diabetes in Yaounde, Cameroon. Diabetic medicine : a journal of the British Diabetic Association. 2015;32(7):886-9.
19. Baumann LC, Frederick N, Betty N, Jospehine E, Agatha N. A demonstration of peer support for Ugandan adults with type 2 diabetes. International journal of behavioral medicine. 2015;22(3):374-83.
20. Prost A, Colbourn T, Seward N, Houweling TAJ, Fottrell E, Lewycka S, et al. Women's groups practising participatory learning and action to improve maternal and newborn health in low-resource settings: A systematic review and meta-analysis. The Lancet. 2013;381(9879):1736-46.
21. Prost A, Colbourn T, Seward N, Azad K, Coomarasamy A, Copas A, et al. Women's groups practising participatory learning and action to improve maternal and

For peer review only - http://bmjopen.bmj.com/site/about/guidelines.xhtml

newborn health in low-resource settings: a systematic review and meta-analysis. The Lancet. 2013;381(9879):1736-46.
22. Ahmed SM, Alam BB, Anwar I, Begum T, Huque R, Khan JAM, et al. Bangladesh Health System Review. Health Systems in Transition. 2015;5(3).
23. Michie S, van Stralen MM, West R. The behaviour change wheel: a new method for characterising and designing behaviour change interventions. Implementation science : IS. 2011;6:42.
24. Michie SJ, M.; Abraham, C.; Lawton, R.; Parker, D.; Walker, A., on behalf of the 'Psychological Theory' Group. Making psychological theory useful for implementing evidence based practice: a consensus approach. Qual Saf Health Care. 2005(14):26-33.
25. Michie S, Richardson M, Johnston M, Abraham C, Francis J, Hardeman W, et al. The behavior change technique taxonomy (v1) of 93 hierarchically clustered techniques: building an international consensus for the reporting of behavior change interventions. Ann Behav Med. 2013;46(1):81-95.
26. Fottrell E, Azad K, Kuddus A, Younes L, Shaha S, Nahar T, et al. The effect of increased coverage of participatory women's groups on neonatal mortality in Bangladesh: a cluster randomized trial. JAMA Pediatrics. 2013;167(9):816-25.
27. Klose T. The contingent valuation method in health care. Health Policy. 1999;47(2):97-123.
28. O'Brien B, Gafni A. When do the "dollars" make sense? Toward a conceptual framework for contingent valuation studies in health care. Med Decis Making. 1996;16(3):288-99.
29. Diener A, O'Brien B, Gafni A. Health care contingent valuation studies: a review and classification of the literature. Health Econ. 1998;7(4):313-26.
30. Hansen KS, Pedrazzoli D, Mbonye A, Clarke S, Cundill B, Magnussen P, et al. Willingness-to-pay for a rapid malaria diagnostic test and artemisinin-based combination therapy from private drug shops in Mukono District, Uganda. Health Policy Plan. 2013;28(2):185-96.
31. Shariful Islam SM, Lechner A, Ferrari U, Seissler J, Holle R, Niessen LW. Mobile phone use and willingness to pay for SMS for diabetes in Bangladesh. J Public Health (Oxf). 2016;38(1):163-9.
32. Russell S, Fox-Rushby J, Arhin D. Willingness and ability to pay for health care: a selection of methods and issues. Health Policy Plan. 1995;10(1):94-101.
33. Byford S, Raftery J. Perspectives in economic evaluation. BMJ. 1998;316(7143):1529-30.
34. Jonsson B. Ten arguments for a societal perspective in the economic evaluation of medical innovations. Eur J Health Econ. 2009;10(4):357-9.
35. Fiedler JL, Villalobos CA, De Mattos AC. An activity-based cost analysis of the Honduras community-based, integrated child care (AIN-C) programme. Health Policy Plan. 2008;23(6):408-27.
36. Johns B, Baltussen R, Hutubessy R. Programme costs in the economic evaluation of health interventions. Cost Eff Resour Alloc. 2003;1(1):1.
37. Batura N, Pulkki-Brannstrom AM, Agrawal P, Bagra A, Haghparast-Bidgoli H, Bozzani F, et al. Collecting and analysing cost data for complex public health trials: reflections on practice. Global health action. 2014;7:23257.
38. Hutton G, Baltussen R. Cost valuation in resource-poor settings. Health Policy Plan. 2005;20(4):252-9.
39. Walker D. Cost and cost-effectiveness guidelines: which ones to use? Health Policy Plan. 2001;16(1):113-21.

For peer review only

For peer review only - http://bmjopen.bmj.com/site/about/guidelines.xhtml

40. Conteh L, Walker D. Cost and unit cost calculations using step-down accounting. Health Policy Plan. 2004;19(2):127-35.
41. Gray AM, Clarke PM, Wolstenholme JL, Wordsworth S. Applied Methods of Cost-effectiveness Analysis in Healthcare (Handbooks in Health Economic Evaluation). Oxford Oxford University Press; 2011.
42. Drummond MF, Sculpher MJ, Claxton K, Stoddart GL, Torrance GW. Methods for the economic evaluation of health care programmes: Oxford university press; 2015.
43. NICE. How NICE Measures Value for Money in Relation to Public Health Interventions. London: NICE (http://www.nice.org.uk) 2013.
44. Tan-Torres Edejer T BR, Adam T, Hutubessy R, Acharya A, Evans DB, Murray CJ. Making choices in health: WHO guide to cost-effectiveness analysis. Geneva: World Health Organization; 2003.
45. Claxton K, Revill P, Sculpher M, Wilkinson T, Cairns J, Briggs A. The Gates Reference Case for Economic Evaluation. The Bill and Melinda Gates Foundation; 2014.
46. Husereau D, Drummond M, Petrou S, Carswell C, Moher D, Greenberg D, et al. Consolidated Health Economic Evaluation Reporting Standards (CHEERS)--explanation and elaboration: a report of the ISPOR Health Economic Evaluation Publication Guidelines Good Reporting Practices Task Force. Value Health. 2013;16(2):231-50.
47. Woods B, Revill P, Sculpher M, Claxton K. Country-Level Cost-Effectiveness Thresholds: Initial Estimates and the Need for Further Research. Value Health. 2016;19(8):929-35.
48. Verguet S, Kim JJ, Jamison DT. Extended Cost-Effectiveness Analysis for Health Policy Assessment: A Tutorial. Pharmacoeconomics. 2016;34(9):913-23.

For peer review only

For peer review only - http://bmjopen.bmj.com/site/about/guidelines.xhtml

For peer review only

For peer review only - http://bmjopen.bmj.com/site/about/guidelines.xhtml

Table 1: Overview of resource use and costs measures included in the economic evaluation of the D-Magic interventions.

Perspective/cost category		Type of costs	Description	Sources	Sample size
Provider	Program/implementing agency	Direct	Costs of implementing mHealth, community mobilisation and HSS interventions	(1) Project accounts of the implementing agencies<br>(2) Interviews with the project staff	N/A
		Indirect	The opportunity cost of volunteer experts attended the mHealth design meetings, donated items, etc.	(1) Project records on numbers of meeting, attendants etc.<br>(2) Published reports on local wage information based on skill category (3) Field offices' inventory information	(1) All meetings held and number of people attended in the meetings will be recorded (2) The list of all equipment in the field offices
	Public healthcare providers	Direct	Changes in utilisation of T2DM and NCD related services at the public health facilities in the study area	(1) Detailed audit and costing study of the health facilities<br>(2) Baseline and end-line cross-sectional surveys (for information on changes in costs of health seeking)	(1) Random sample of health facilities at different levels in both intervention and control areas<br>(2) All participants in the study
		Indirect	The opportunity cost of the time spent by the health care providers attending HSS meetings	(1) Project records on numbers of meeting, attendants etc.<br>(2) Published reports on local wage information based on skill category	All meetings held and number of people attended in the meetings will be recorded
Participants/households		Direct	Household expenditure on food and non-food	Household consumption expenditure survey	A random sample of 300 households in the study area
			Costs of health seeking for the participants and their households	Baseline and end-line cross-sectional surveys	All participants in the study
		Indirect	Opportunity cost of participation in the groups	Group participants survey	A random sample of 312 group participants (both male and female)

* HSS: health system strengthening, T2DM: type 2 Diabetes mellitus, NCD: non-communicable diseases

For peer review only - http://bmjopen.bmj.com/site/about/guidelines.xhtml

For peer review only

For peer review only - http://bmjopen.bmj.com/site/about/guidelines.xhtml

CHEERS Tool

Section/item		Item no.	Recommendation	Response
Title and abstract	Title	1	Identify the study as an economic evaluation, or use more specific terms such as ''cost-effectiveness analysis'' and describe the interventions compared.	Yes
	Abstract	2	Provide a structured summary of objectives, perspective, setting, methods (including study design and inputs), results (including base-case and uncertainty analyses), and conclusions.	Yes
Introduction	Background and objectives	3a	Provide an explicit statement of the broader context for the study.	Yes
		3b	Present the study question and its relevance for health policy or practice decisions.	Yes
Methods	Target population and subgroups	4	Describe characteristics of the base-case population and subgroups analyzed including why they were chosen.	Yes
	Setting and location	5	State relevant aspects of the system(s) in which the decision(s) need(s) to be made.	Yes
	Study perspective	6	Describe the perspective of the study and relate this to the costs being evaluated.	Yes
	Comparators	7	Describe the interventions or strategies being compared and state why they were chosen.	Yes
	Time horizon	8	State the time horizon(s) over which costs and consequences are being evaluated and say why appropriate.	Yes
	Discount rate	9	Report the choice of discount rate(s) used for costs and outcomes and say why appropriate.	Yes
	Choice of health outcomes	10	Describe what outcomes were used as the measure(s) of benefit in the evaluation and their relevance for the type of analysis performed.	Yes
	Measurement of effectiveness	11 a)	Single study–based estimates: Describe fully the design features of the single effectiveness study and why the single study was a sufficient source of clinical effectiveness data.	Yes
		11 b)	Synthesis-based estimates: Describe fully the methods used for the identification of included studies and synthesis of clinical effectiveness data.	n/a
	Measurement and valuation of preference based outcomes	12	If applicable, describe the population and methods used to elicit preferences for outcomes.	n/a
	Estimating resources and costs	13 a)	Single study–based economic evaluation: Describe approaches used to estimate resource use associated with the alternative interventions. Describe primary or secondary research methods for valuing each resource item in terms of its unit cost. Describe any adjustments made to approximate to opportunity costs.	Yes
		13 b	Model-based economic evaluation: Describe approaches and data sources used to estimate resource use associated with model health states. Describe primary or secondary research methods for valuing each resource item in terms of its unit cost. Describe any adjustments made to approximate to opportunity costs.	n/a

	Currency, price date, and conversion	14	Report the dates of the estimated resource quantities and unit costs. Describe methods for adjusting estimated unit costs to the year of reported costs if necessary. Describe methods for converting costs into a common currency base and the exchange rate.	Yes
	Choice of model	15	Describe and give reasons for the specific type of decision-analytic model used. Providing a figure to show model structure is strongly recommended.	n/a
	Assumptions	16	Describe all structural or other assumptions underpinning the decision-analytic model.	n/a
	Analytical methods	17	Describe all analytic methods supporting the evaluation. This could include methods for dealing with skewed, missing, or censored data; extrapolation methods; methods for pooling data; approaches to validate or make adjustments (e.g., half-cycle corrections) to a model; and methods for handling population heterogeneity and uncertainty.	Yes
Results	Study parameters	18	Report the values, ranges, references, and if used, probability distributions for all parameters. Report reasons or sources for distributions used to represent uncertainty where appropriate. Providing a table to show the input values is strongly recommended.	Not possible in the protocol although the process is described
	Incremental costs and outcomes	19	For each intervention, report mean values for the main categories of estimated costs and outcomes of interest, as well as mean differences between the comparator groups. If applicable, report incremental cost-effectiveness ratios.	Not possible in the protocol although the process is described
	Characterising uncertainty	20 a	Single study–based economic evaluation: Describe the effects of sampling uncertainty for estimated incremental cost, incremental effectiveness, and incremental cost-effectiveness, together with the impact of methodological assumptions (such as discount rate, study perspective).	Not possible in the protocol although the process is described
		20 b	Model-based economic evaluation: Describe the effects on the results of uncertainty for all input parameters, and uncertainty related to the structure of the model and assumptions.	n/a
	Characterising heterogeneity	21	If applicable, report differences in costs, outcomes, or cost-effectiveness that can be explained by variations between subgroups of patients with different baseline characteristics or other observed variability in effects that are not reducible by more information.	Not possible in the protocol although the process is described
Discussion	Study findings, limitations, generalisability, and current knowledge	22	Summarize key study findings and describe how they support the conclusions reached. Discuss limitations and the generalizability of the findings and how the findings fit with current knowledge	Not possible in the protocol although the process is described
Other	Source of funding	23	Describe how the study was funded and the role of the funder in the identification, design, conduct, and reporting of the analysis. Describe other nonmonetary sources of support.	Yes
	Conflicts of interest	24	Describe any potential for conflict of interest among study contributors in accordance with journal policy. In the absence of a journal policy, we recommend authors comply with International Committee of Medical Journal Editors' recommendations.	Yes

BMJ Open

Protocol of economic evaluation and equity impact of m-Health and community groups for prevention and control of diabetes in rural Bangladesh in a three-arm cluster randomised controlled trial

Journal:	*BMJ Open*
Manuscript ID	bmjopen-2018-022035.R2
Article Type:	Protocol
Date Submitted by the Author:	25-Jun-2018
Complete List of Authors:	Haghparast-Bidgoli, Hassan; University College London, Institute for Global Health<br>Shaha, Sanjit ; Diabetic Association of Bangladesh<br>Kuddus, Abdul; Diabetic Association of Bangladesh, Perinatal Care Project<br>Chowdhury, Md.Alimul Reza ; Diabetic Association of Bangladesh<br>Jennings, Hannah ; University College London, Institute for Global Health<br>Ahmed, Naveed; Diabetic Association of Bangladesh<br>Morrison, Joanna; University College London, Institute for Global Health<br>Akter, Kohenour; Diabetic Association of Bangladesh<br>Nahar, Badrun ; Diabetic Association of Bangladesh<br>Nahar, Tasmin ; Diabetic Association of Bangladesh<br>King, C.; UCL, Institute for Global Health<br>Skordis-Worrall, Jolene; University College London Medical School,<br>Batura, Neha; University College London, Institute for Global Health<br>Khan, Jahangir; Liverpool School of Tropical Medicine, Health Economics<br>Mansaray, Anthony ; ICAP Sierra Leone<br>Hunter, Rachael; University College London, Research Dept of Primary Care and Population Health<br>Azad Khan, A.K. ; Diabetic Association of Bangladesh<br>Costello, Anthony ; University College London, Institute for Global Health; World Health Organisation<br>Azad, Kishwar; Diabetic Association of Bangladesh,<br>Fottrell, Edward; University College London, Institute for Global Health
**Primary Subject Heading**:	Health economics
Secondary Subject Heading:	Diabetes and endocrinology, Global health, Health policy, Health services research, Public health
Keywords:	HEALTH ECONOMICS, DIABETES & ENDOCRINOLOGY, Health policy < HEALTH SERVICES ADMINISTRATION & MANAGEMENT

SCHOLARONE™
Manuscripts

For peer review only

Protocol of economic evaluation and equity impact of m-Health and community groups for prevention and control of diabetes in rural Bangladesh in a three-arm cluster randomised controlled trial

Hassan Haghparast-Bidgoli[1*], Sanjit Kumar Shaha[2], Abdul Kuddus[2], Md.Alimul Reza Chowdhury[2], Hannah Jennings[1], Naveed Ahmed[2], Joanna Morrison[1], Kohenour Akter[2], Badrun Nahar[2], Tasmin Nahar[2], Carina King[1], Jolene Skordis[1], Neha Batura[1], Jahangir A. M. Khan[4], Anthony Mansaray[5], Rachael Hunter[6], A.K. Azad Khan[2], Anthony Costello[1, 3], Kishwar Azad[2], Edward Fottrell[1]

1 University College London, Institute for Global Health, London, United Kingdom
2 Diabetic Association of Bangladesh, Dhaka, Bangladesh
3 World Health Organisation, Geneva, Switzerland
4 Liverpool School of Tropical Medicine, Liverpool, United Kingdom
5 ICAP Sierra Leone, Sierra Leone.
6 University College London, Institute of Epidemiology & Health, London, United Kingdom

*Corresponding author
Hassan Haghparast-Bidgoli
Address: University College London, Institute for Global Health, 30 Guilford Street, London WC1N 1EH, UK
Email: h.haghparast-bidgoli@ucl.ac.uk
Tel: +44 (0) 20 7905 2779

Word count: 4437

For peer review only

Abstract

Introduction: Type 2 Diabetes mellitus (T2DM) is one of the leading causes of death and disability worldwide, generating substantial economic burden for people with diabetes and their families, and to health systems and national economies. Bangladesh has one of the largest numbers of adults with diabetes in the South Asian region. This paper describes the planned economic evaluation of a three-arm cluster randomised control trial of mHealth and community mobilisation interventions to prevent and control T2DM and non-communicable diseases' risk factors in rural Bangladesh (D-Magic trial).

Methods and Analysis: The economic evaluation will be conducted as a within-trial analysis to evaluate the incremental costs and health outcomes of mHealth and community mobilisation interventions compared to the status quo. The analyses will be conducted from a societal perspective, assessing the economic impact for all parties affected by the interventions, including implementing agencies (program costs), health care providers, and participants and their households. Incremental cost-effectiveness ratios (ICERs) will be calculated in terms of cost per case of intermediate hyperglycaemia and T2DM prevented and cost per case of diabetes prevented among individuals with intermediate hyperglycaemia at baseline, and cost-per mm Hg reduction in systolic blood pressure. In addition to ICERs, the economic evaluation will be presented as a cost-consequence analysis where the incremental costs and all statistically significant outcomes will be listed separately. Robustness of the results will be assessed through sensitivity analyses. In addition, an analysis of equity impact of the interventions will be conducted.

Ethics and Dissemination: The approval to conduct the study was obtained by the University College London Research Ethics Committee (4766/002) and by the Ethical Review Committee of the Diabetic Association of Bangladesh (BADAS-ERC/EC/t5100246). The findings of this study will be disseminated through different means within academia and the wider policy sphere.

Trial registration: ISRCTN41083256. Registered 30/03/2016

For peer review only

Strengths and limitations of this study

- This protocol paper reports planned data collection and analyses alongside a complex public health trial to ensure transparency.
- This protocol can assist in designing mHealth and community mobilisation through Participatory Learning and Action Approach interventions for the prevention of diabetes and mitigation of NCDs risk factors.
- The protocol, and planned analysis and reporting follow recommended guidelines to design and report economic evaluations.
- An equity impact analysis and contingent valuation study will be conducted alongside the study.
- The study is powered to assess the cost-effectiveness of each intervention against control only. The study is not powered to test the differences between the mHealth and community mobilisation interventions. Therefore, it will be possible to estimate incremental cost and effect of each intervention compared to the control only.

For peer review only

For peer review only - http://bmjopen.bmj.com/site/about/guidelines.xhtml

BACKGROUND

Burden of diabetes mellitus

Diabetes mellitus, mainly type 2, is a one of the leading causes of death and disability worldwide. It is estimated that around 415 million people worldwide, or 9% of adults aged 20-79 have diabetes, with about 75% of people living with diabetes residing in low- and middle-income countries (LMICs) (1). It is predicted that by 2040, one in 10 adults will have diabetes, unless preventive efforts are undertaken (1). Diabetes, if not managed properly, can lead to several complications, such as heart attack, kidney failure, leg amputation, vision loss and several other long-term consequences, that impact significantly on quality of life and cause premature death (1, 2). Diabetes and its complications create a substantial economic burden for people with diabetes and their families, and for health systems and national economies through direct medical costs and productivity loss (2). It is reported that in many countries between 5% and 20% of total health expenditure are spent on diabetes (1). This is in addition to the large financial burden on individuals and their families due to the cost of seeking care.

Bangladesh has the second largest number of adults with diabetes in the South Asian region, with around 7 million adults aged 20-79 years with diabetes (1). The prevalence of diabetes among adults (20-79 years old) in Bangladesh is estimated to be around 8.5 % (1, 2), with a three to four-fold increase since the 1990s (3, 4). It is predicted that prevalence of diabetes in Bangladesh will reach 23.6% in men and 33.5% in women by 2030, unless preventive efforts are undertaken (3). The increasingly high incidence of diabetes in Bangladesh has had a significant economic burden, in particular for people with diabetes, their families and the country's health care system. It is estimated that the annual health expenditure for diabetes is around US$218 million, where most of the costs born by the families (5).

Underlying the increasing prevalence of diabetes, globally and in Bangladesh, are complex genetic, environmental and lifestyle factors, including changes in dietary habits and increases in risk factors like smoking and physical inactivity (2). Effective interventions are available to prevent type 2 Diabetes mellitus (T2DM), and to prevent the complications and premature death from diabetes (2). The evidence from LMICs shows that lifestyle and other non-pharmacological interventions can prevent and delay the onset of T2DM and its complications (6). However, there is a lack of cost effective programs designed specifically for Bangladeshi populations, taking into account their contextual needs and resources.

The Bangladesh D-Magic Trial

The Bangladesh D-magic is a three-arm cluster randomised control trial (cRCT) of mHealth and community mobilisation interventions, conducted in four rural upazilas in Faridpur district, Bangladesh. The trial aims to assess the effectiveness and cost-effectiveness of the interventions in the prevention and control of T2DM and NCD risk factors in rural Bangladesh. In the D-magic trial, the clusters (96 villages) were

For peer review only - http://bmjopen.bmj.com/site/about/guidelines.xhtml

randomised to receive either the mHealth intervention, the community mobilisation intervention, or be in the control arm (32 in each). In the mHealth intervention, individuals receive voice messages about prevention and control of NCD risk factors and T2DM on their mobile phone. In the community mobilisation intervention, a trained facilitator initiates a series of diabetes and NCD risk-factor focused monthly group meetings for men and women, working through a participatory learning and action (PLA) cycle by which group members themselves identify, prioritise and tackle problems associated with T2DM and its risk factors. In addition, all study areas (both intervention and control) clusters receive health system strengthening activities, which include the training/refresher training of health care workers working in the community and health facilities, in the prevention, diagnosis and treatment of T2DM, as well as the development of essential equipment inventories.

Details of the D-Magic trial are described elsewhere (7). This protocol paper aims to fully describe the methodology for the economic evaluation of the trial.

Economic evaluations of community and mHealth interventions for prevention and control of diabetes

There is evidence that mHealth programs can have a positive impact on behaviour change and prevention and control of diabetes and NCDs in high-risk populations (8-11). However, there is little information on the cost and cost-effectiveness of mHealth interventions for the prevention and control of NCDs (12-14). Two recent systematic reviews of the economic evidence of mHealth (13), and mHealth for diabetes prevention and control (14), have shown that there are a handful of NCD and diabetes interventions that have reported cost and cost effectiveness evidence. Nearly all of these studies have been conducted in high-income settings. The majority of these studies report that mHealth interventions are cost-effective or cost saving, though the quality of reported evidence was not satisfactory in some of cases (13, 14).

Similarly, although there is some evidence on effectiveness of community-based interventions in the management of T2DM in low-income settings (15-19), there is little evidence on how cost effective these interventions might be (14). A recent review (14) has identified 10 community-based interventions on preventing and controlling diabetes. These interventions, which are largely implemented in high-income settings, have reported that community-based interventions are cost-effective or cost-saving approaches in the management of T2DM (14).

The current study will be the first to assess the cost-effectiveness of community mobilisation through PLA in the prevention of T2DM. This approach has previously been shown to be highly cost-effective in improving maternal and newborn health (20, 21). This study will also contribute to the evidence on cost-effectiveness of mHealth interventions for preventing T2DM in LMIC settings.

Aim and objectives

For peer review only - http://bmjopen.bmj.com/site/about/guidelines.xhtml

The D-Magic economic evaluation aims to measure the cost-effectiveness of mHealth and participatory community group interventions to prevent and to control T2DM and NCD risk factors in rural Bangladesh from a societal perspective.

The specific objectives of the D-Magic economic evaluation are:

1. To estimate the costs of setting up and implementing the mHealth and participatory community group interventions as well as health system strengthening (HSS) activities;
2. To calculate the costs to the healthcare system, of increased care-seeking (i.e. diagnosis and treatment) for T2DM and other NCDs, as a result of the D-Magic interventions;
3. To measure costs associated to the intervention participants and their households of changes in diabetes or other NCD-related diagnosis and management care-seeking costs as well as any costs associate with changes in diet and other life-style behaviours, as a result of the D-Magic interventions;
4. To present the incremental costs and outcomes of the interventions as a cost-consequence analysis;
5. To calculate the incremental cost-effectiveness of the mHealth and community mobilisation interventions combined with HSS activities, as compared to HSS activities alone, where all new HSS activities are delivered in addition to the existing government programmes.

In addition to the above-mentioned objectives, the equity impact of the mHealth and community mobilisation interventions will be assessed.

METHODS

Study setting and population

The study setting for the D-Magic trial is Faridpur district, located south of Dhaka. The district has a population of over 1.7 million people in a high-density area of just over 2000 square kilometres. It is primarily an agricultural economy, with the main crops being jute and rice. Like in the rest of Bangladesh, healthcare is provided at three levels: primary care is provided at Community Clinics and at Union Health and Family Welfare Centres; secondary level care providers (both in- and out-patient services) are sub-district (Upazilla) health complexes and hospitals; and tertiary care is provided at district hospitals and medical college hospitals (22). Private and informal service providers are also present in the area, with the informal sector being the main provider in rural areas (22), Faridpur district included. Inadequate and inequitable access to services, shortages of skilled health care providers, short supplies of medicines and poor quality and low responsiveness of services are the main challenges faced by the Bangladesh healthcare system (22), and remains a challenge in Faridpur district too.

The study population for the D-Magic study are men and non-pregnant women who are aged 30 years or more and permanent residents (i.e. lived there for more than 6 month) of 96 villages (clusters) in four rural upazillas – Nagarkanda, Boalmari, Saltha and Madhukhali – in Faridpur district.

For peer review only

Trial design

The D-Magic trial is a three-arm cRCT which is implemented in four rural upazilas in Faridpur district, Bangladesh. In D-Magic trial, 96 clusters (villages) were randomised to receive either the mHealth intervention, the community mobilisation intervention, or be in the control arm. All three arms receive a number of health system strengthening activities. The interventions implementation was started in July 2016 and was completed by end of December 2017 and all data collection was completed in June 2018.

Detailed information on the randomisation and participants recruitment process, trial time-line, and a full description of the interventions is presented elsewhere (7). A brief description of the mHealth, community mobilisation and health system strengthening interventions are presented in the following sections.

mHealth intervention

The mHealth intervention involves free of charge voice messages about the prevention and control of T2DM and NCD risk factors sent twice-weekly to the individual's mobile phone during a 14-month period. The intervention and the messages' content were developed based on findings from baseline formative research in the study area, and application of the behaviour change theories the COM-B (Capability, Opportunity, Motivation, Behaviour) model for understanding behaviour (23) and the Theoretical Domains Framework (TDF) to encourage change (24, 25). The mHealth intervention is available to all individuals who have access to a mobile phone and registered to receive the messages by providing their mobile number to the intervention community recruiters.

Community Mobilisation intervention

The community mobilisation intervention involves initiation and facilitation of separate male and female participatory groups, with approximately 20 members in each. The intervention is an adaptation of a participatory women's groups intervention implemented in South Asia (including Bangladesh) and Sub-Saharan African settings and shown to be effective and cost effective at reducing neonatal mortality (21, 26). The groups progress through a series of 18 monthly meetings following the four phases of PLA. During phase 1, participants identify and prioritise factors that affect their health, particularly those increasing their risk of developing or failing to manage T2DM; in phase 2, the participants and their community come up with feasible strategies that can be implemented to address the problems identified in phase 1; during phase 3, they implement these strategies; in phase 4 they evaluate the strategies they have implemented. The groups are run by salaried facilitators, who are recruited from the study areas and have a minimum of higher secondary school education. The facilitators undertook one week's training on group facilitation and basic health messages related to NCD prevention and control, in particular T2DM. They also have received refresher training during the course of the interventions.

Health Systems Strengthening (HSS)

For peer review only - http://bmjopen.bmj.com/site/about/guidelines.xhtml

A series of health system strengthening activities are carried out in all study areas (both intervention and control clusters). These activities, which are tailored according to the mapping of health care providers in the project area in the formative phase of the project, included the training of mainly informal health care workers in the community and distributing educational materials among formal and informal providers in prevention, diagnosis and treatment of T2DM, as well as the development of essential equipment inventories.

Measurement of health outcomes/effectiveness

The D-Magic trial will test the effect of the community mobilisation intervention and mHealth intervention relative to the control and does not directly compare the effects of each intervention relative to the other. As this is a cluster-randomised trial, the outcomes will be measured among individuals (permanent residents) who live in the intervention clusters, irrespective of whether they took part in groups or received mHealth messages. Analysis of the outcomes will be by intention to treat at the individual and cluster level as appropriate. Moreover, participants with missing data on the primary outcomes will be excluded from primary outcome analysis (7).

Primary outcome

The D-Magic trial has two primary outcomes: (1) combined prevalence of intermediate hyperglycaemia (i.e. impaired fasting glucose or impaired glucose tolerance) and T2DM, and (2) cumulative two-year incidence of T2DM among individuals identified with intermediate hyperglycaemia at the start of the trial (7). Using the prevalence data, the number of intermediate hyperglycaemia and diabetes mellitus cases prevented, and the number of diabetes cases prevented among individuals with intermediate hyperglycaemia at baseline will be calculated as the difference between the expected and the actual number of cases using the adjusted odds ratio relative to the control population.

Secondary outcomes

The trial has a number of secondary outcomes including diastolic and systolic blood pressure, prevalence of hypertension, body mass index, prevalence of overweight and obesity, prevalence of abdominal obesity, health-related quality of life, and psychological distress among self-reported diabetics (7).

Comparison will be made between the interventions (mHealth and community mobilisation) and control (HSS activities only) to estimate incremental cost-effectiveness ratios (ICERs) for the primary outcomes; in terms of cost per case of intermediate hyperglycaemia and diabetes mellitus prevented and cost per case of diabetes prevented among individuals with intermediate hyperglycaemia at baseline, and for some of the secondary outcomes such as cost- per mm Hg reduction in systolic BP. ICER will be conducted if a significant impact on the outcomes is observed. Moreover, all costs and (statistically significant) outcomes, both primary and secondary, will be presented separately in a cost-consequence analysis.

For peer review only - http://bmjopen.bmj.com/site/about/guidelines.xhtml
For peer review only

Willingness to pay/Contingent Valuation study

We will conduct a willingness to pay (WTP) study in order to elicit maximum monthly amount of money each participant or household would be willing to pay if an m-Health service on diabetes and NCD risk factors' prevention and management (i.e. weekly voice messages) was available.

WTP studies are widely used in order to elicit monetary value of a service or good not available in the market (27-30). In the Bangladesh context, Islam et al (2015) in a recent study estimated WTP of T2DM patients for receiving messages for increasing adherence to treatment (31). Similar to Islam et al (2015), we will use an open-ended contingent valuation (CV) method (27, 30, 32), asking participants through an open-ended question how much they would be willing to pay monthly to receive voice messages related to diabetes and NCD risk factors' prevention and management, if such a service was available. Open-ended CV is a more flexible approach and avoids starting point bias or range bias introduced by other methods such bidding game and payment cards (27). The theoretical framework defined by O'Brien and Gafni (28) for CV studies will be used to design the study.

WTP questions will be asked from all the participants at the end-line impact evaluation survey. WTP values will be compared across different groups, for example, based on exposure to the interventions (i.e. among those exposed to mHealth, or exposed to participatory groups, and participants who have not been exposed to either of these interventions) or health condition (people diagnosed with diabetes or other NCDs and others). In addition, for each participant, detailed individual and household level socio-economic characteristics will be collected to examine the extent to which WTP values will vary by socio-economic status of participants.

Equity impact of the D-Magic interventions

In addition to measuring efficiency/cost-effectiveness of the D-Magic interventions, an analysis of equity impact of the interventions will be conducted to assess whether impacts/gains from the intervention are equitably shared among the target population. This will be done through sub-group analyses of the primary and secondary outcomes based on the socio-economic status of the target population.

Identification, measurement and valuation of resource use

The cost-effectiveness and cost-consequence of the D-Magic interventions will be measured from a societal perspective (33, 34); measuring the economic impact for all parties affected by the interventions, including implementing agency (or program costs), public healthcare providers (at both local and national levels) and users, who are the intervention participants and their households. The following sections provide a detailed description of the proposed methods for measuring and valuing programme costs, healthcare provider costs and participants/household costs. Programme costs include those incurred by the implementing agency or program provider i.e. Diabetic Association of Bangladesh (BADAS). The healthcare provider costs are those incurred by the government health facilities in the study area including community

For peer review only - http://bmjopen.bmj.com/site/about/guidelines.xhtml

clinics, Health & Family Welfare centres and Upazila Health Complex/hospitals. The household or user costs include those incurred by participants and their households. Table 1 provides an overview of the financial and economic costs to be employed in the economic evaluation of the D-Magic interventions.

Programme-related costs

Direct and indirect costs of designing and implementing mHealth, community mobilisation and health system strengthening interventions will be estimated using a combination of activity-based costing (35) and ingredients approach (36).

Financial or expenditure data

Programme costs are mainly financial or accounting costs, which are collected prospectively from the project accounts or expenditure records of the implementing partner and entered (generally, on an annual basis) to an MS Excel data capture tool. The tool contains different sections/worksheets that will allow the categorization of costs into line items (i.e. staff, materials, capital, and joint costs), start up and implementation costs of the interventions, and costs associated with the different programme components, i.e. m-Health, community mobilization, HSS, and monitoring and evaluation. Key informant interviews with project leads and monthly/quarterly staff time sheets will be used to allocate joint costs between the programme components. The summary worksheets in the cost data capture tool present the costs by programme component (e.g. mHealth, community mobilisation and HSS), summarise the total cost data per intervention, allows effect data to be entered and estimates the cost-effectiveness results.

Donated items and opportunity costs

Some items, such as donated items and volunteer time, are not captured in the accounting system and need to be converted to economic costs using their market value and then entered into the data capture tool (37-39). Potential donated items are equipment donated by the implementing agency (i.e. purchased by previous projects and used in the D-Magic project). The donated items will be identified through key informant interviews with the project leads.

The majority of volunteer time is related to designing messages for the mHealth intervention, where several meetings were held with experts who were volunteers. Detailed information regarding these meetings, including the number of meetings, their duration, and the participants is being documented by the project. The opportunity cost of the time invested by the experts will be measured as a proportion of their salary or a salary equivalent using published national/local wage rate reports.

Public healthcare providers costs

The D-Magic project is likely (at least in the short term) to increase seeking care for diabetes, other NCDs and NCD risk factors such as hypertension, particularly demand for services such as testing and treatment for hypertension, diabetes and pre-diabetes, or seeking advice or treatment for weight control. In addition, there is a time

For peer review only - http://bmjopen.bmj.com/site/about/guidelines.xhtml

(opportunity) cost of direct involvement in the HSS activities for the healthcare providers (Table 1).

Cost of changes in demand for services

The costs to public healthcare providers in the project area due to increased (or any changes in) demand for their services will be estimated. A mapping of the health care providers in the study area has been completed and 20 functional governmental health care facilities, at different levels, have been identified. These facilities included 14 Community Clinics, 3 Health & Family Welfare centres and 3 Upazila Health Complex/Hospitals. A sample of 11 health facilities including 8 community clinics and 2 Health & Family Welfare centres (with equal numbers in control and intervention clusters), as well as one Upazila Health Complex/Hospital (which cover both control and intervention areas) were selected for baseline audits of diabetes and NCD services, estimating their resource utilisations and unit costs. Similar audits and cost data collection will be conducted for the same facilities post-intervention in order to assess the changes in NCD service utilisation attributable to the D-Magic interventions. This data will be complemented by health seeking behaviour information collected from the study participants in control and interventions clusters at the D-Magic end-line impact evaluation survey. Differences in service utilisation between intervention and control areas will be attributed to the D-Magic interventions.

A simple audit and cost-capture tool was developed for facility data collection and piloted with facilities at different levels. Data from the cost-capture tool will be complemented by the existing data from the facility reports, and published data. Costs of services provided by the facilities will be estimated using a step-down approach (40).

Any change in demand for services provided in the facilities other than those mentioned above and the services not covered by them, in intervention areas compared to the control, will be identified during the trial's routine monitoring and end-line impact evaluation survey. Any cost of that change in demand will be calculated using published data on the unit costs of those services.

Opportunity cost of HSS activities

Moreover, as discussed earlier, HSS activities include several training sessions for health care providers in prevention, diagnosis and treatment of T2DM. Information on the number of meetings, their duration and participation is being documented by the project. The opportunity cost of the time spent by the providers will be measured as a proportion of their salary for formal providers or as a salary equivalent, for informal providers.

Participants and their household costs

D-Magic interventions may influence participants and their households' costs in a number of ways. These include changes in health seeking behaviour, and changes in household lifestyles that might affect food and non-food consumption patterns and

For peer review only - http://bmjopen.bmj.com/site/about/guidelines.xhtml

spending as well as time spent engaging in physical activities. It also includes the time (opportunity) cost of participation in the PLA group meetings and participating in the actions taken by the groups (Table 1).

Health care seeking costs

D-Magic may increase the participants and their households' seeking advice and care from both formal and informal providers for testing and treatment for hypertension, diabetes, pre-diabetes and other NCDs. Information on costs of care seeking are collected from all the participants recruited in the project, at the baseline and end-line evaluation surveys. This information will be complemented by the data collected from the household consumption expenditure survey. The difference in spending for the participants and their households will be calculated and compared between intervention and control areas.

Changes in household food and non-food expenditure

Changes in food and non-food expenditure will be captured in a comprehensive household consumption and expenditure survey. The survey will be conducted on a random sub-sample of 300 households (100 per trial arm) at the end of intervention period. The changes in the expenditure will be compared between interventions and control areas.

Opportunity cost of participation in the interventions

Participating in the PLA group meetings incur some costs to the participants and their family. These costs include the direct costs (e.g. cost of getting to the group) and time cost of group participation (e.g. travel time and time spent in the group) and participating in the actions taken by the group, or changing health and lifestyle behaviours. Information on the potential direct and time costs will be collected through a sub-sample survey of 312 group participants (both male and female), randomly selected. Sample size for the survey was primarily calculated to give sufficiently accurate estimate of group participants' characteristics.

Cost-effectiveness and cost-consequence analyses

Economic evaluation will be conducted as a within-trial analysis using the intention-to-treat results, and will be presented in terms of ICERs, calculated as the difference in total costs of mHealth and community mobilisation interventions (plus HSS activities) versus HSS activities only (or control), divided by the difference in mean effects of each interventions versus control (41) (42). As mentioned previously, ICERs will be evaluated in terms of cost per case of intermediate hyperglycaemia and T2DM prevented and cost per case of T2DM prevented among individuals with intermediate hyperglycaemia at baseline, and for some of the secondary outcomes such as cost- per mm Hg reduction in systolic BP. In addition to ICERs, the economic evaluation will be presented as a cost-consequence analysis where the incremental costs and all statistically significant outcomes will be listed separately, allowing policymakers to compare the costs and all impacts/gains of the D-Magic

For peer review only - http://bmjopen.bmj.com/site/about/guidelines.xhtml

interventions. Cost-consequence analysis has been recommended for complex public health interventions, such as D-Magic, that have multiple health and non-health impacts, which are difficult to measure in a common outcome unit (42, 43).

All costs will be presented in 2017 prices in Bangladeshi Taka and International Dollars (INT$). All costs will be adjusted for inflation using the Bangladeshi Consumer Price Index (CPI) and will be converted to 2017 INT$ using the Purchasing Power Parity (PPP) conversion factor for Bangladesh. Moreover, both costs and outcomes will be discounted using a standard discount rate of 3%, as recommended by WHO-CHOICE (44) and the Gates Reference Case for Economic Evaluation (45). The impact of uncertainty in key parameters on the cost-effectiveness results will be assessed through a series of deterministic and probabilistic sensitivity analyses. Reporting of the study design, analytical methods and findings will follow the Consolidated Health Economic Evaluation Reporting Standards (CHEERS) statement (46). The D-Magic interventions will be judged to be cost-effective and affordable (though, indirectly) against the WHO-CHOICE recommendation (44), as well as recently developed cost-effectiveness thresholds (47).

The possibility of conducting an extended cost-effectiveness analysis (48) alongside modeling national scale up of the D-Magic interventions will be explored. Moreover, we will explore the possibility of running a long-term cost-effectiveness analysis using decision analytical modelling based on the relevant outcomes such as systolic blood pressure, body mass index, etc. (if statistically significant) to predict future economic impacts from implementing the D-Magic interventions on the target population.

Strengths and limitations of the study

In order to increase transparency and minimize bias, publication and peer-review of economic evaluation protocols is encouraged. This study reports planned data collection and analyses alongside a complex public health trial. The study will contribute to the scarce cost-effectiveness evidence on mHealth and community mobilisation interventions for preventing diabetes and NCDs.

Furthermore, adopting a cost-consequence analysis approach makes it possible to report all health and non-health impacts of the D-Magic interventions, in addition to ICERs, which can assist policy makers to make informed decisions in designing or implementing similar complex interventions. Incorporating equity impact analysis and contingent valuation are other strengths of this study, which provide useful information for future scale-up of the interventions.

The study design has one limitation. The D-Magic trial is not powered to test the differences between the mHealth and community mobilisation interventions due to the large sample size required and the resources available for the trial. However, the possibility of a direct comparison between the two interventions will be explored.

Patient and public involvement

For peer review only - http://bmjopen.bmj.com/site/about/guidelines.xhtml

Patients and public were not involved in the process of this study. Patients and public will be informed of the study results via peer-reviewed journals, conference and local dissemination meetings.

Contributors

HHB, EF, AK, HJ, JM, CK, JS, NB, JK, RH, AKAK, AC and KA contributed to the study design; HHB, EF, CK contributed to the statistical analyses; SKS, MARC, NA, KA, BN and TN contributed to data acquisition; AM has contributed to the literature review and drafting the paper; HHB was responsible for the initial drafting of this manuscript; all authors contributed to the review of this manuscript and provided comments. All authors read and approved the final manuscript.

Funding

The trial is funded by the Medical Research Council UK (MR/M016501/1) under the Global Alliance for Chronic Diseases (GACD) Diabetes Programme.

Competing interests

The authors declare they have no competing interests.

Ethics and Dissemination

The D-Magic trial has been approved by the University College London Research Ethics Committee (4766/002) and by the Ethical Review Committee of the Diabetic Association of Bangladesh (BADAS-ERC/EC/t5100246). The trial has been registered and assigned an International Standard Randomised Controlled Trial Number (ISRCTN41083256). Informed cluster-level consent from village/community leaders, and individual informed consent was obtained from all participants in the trial prior to their participation in the study. The findings of this study will be disseminated through different means within academia and the wider policy sphere.

References

1. International Diabetes Federation. Diabetes Atlas: Seventh Edition. Brussels, Belgium: International Diabetes Federation; 2015.
2. World Health Organization. Global report on diabetes. Geneva, Switzerland World Health Organization; 2016.
3. Rahman MM, Akter S, Jung J, Rahman MS, Sultana P. Trend, projection, and appropriate body mass index cut-off point for diabetes and hypertension in Bangladesh. Diabetes research and clinical practice. 2017;126:43-53.
4. Saquib N, Saquib J, Ahmed T, Khanam MA, Cullen MR. Cardiovascular diseases and type 2 diabetes in Bangladesh: a systematic review and meta-analysis of studies between 1995 and 2010. BMC public health. 2012;12:434.
5. Sarker AR, Sultana M. Health and economic burden of diabetes in Bangladesh: Priorities for attention and control. Journal of diabetes. 2017;9(12):1118-9.
6. Rawal LB, Tapp RJ, Williams ED, Chan C, Yasin S, Oldenburg B. Prevention of type 2 diabetes and its complications in developing countries: a review. International journal of behavioral medicine. 2012;19(2):121-33.
7. Fottrell E, Jennings H, Kuddus A, Ahmed N, Morrison J, Akter K, et al. The effect of community groups and mobile phone messages on the prevention and

For peer review only - http://bmjopen.bmj.com/site/about/guidelines.xhtml

control of diabetes in rural Bangladesh: study protocol for a three-arm cluster randomised controlled trial. Trials. 2016;17(1):600.
8. Hamine S, Gerth-Guyette E, Faulx D, Green BB, Ginsburg AS. Impact of mHealth chronic disease management on treatment adherence and patient outcomes: a systematic review. Journal of medical Internet research. 2015;17(2):e52.
9. Shaw R, Bosworth H. Short message service (SMS) text messaging as an intervention medium for weight loss: A literature review. Health informatics journal. 2012;18(4):235-50.
10. Shetty AS, Chamukuttan S, Nanditha A, Raj RK, Ramachandran A. Reinforcement of adherence to prescription recommendations in Asian Indian diabetes patients using short message service (SMS)--a pilot study. The Journal of the Association of Physicians of India. 2011;59:711-4.
11. Ramachandran A, Snehalatha C, Ram J, Selvam S, Simon M, Nanditha A, et al. Effectiveness of mobile phone messaging in prevention of type 2 diabetes by lifestyle modification in men in India: a prospective, parallel-group, randomised controlled trial. The lancet Diabetes & endocrinology. 2013;1(3):191-8.
12. Stephani V OD, Quentin W,. Does mHealth contribute to improved care for people with non-communicable diseases in developing countries? A systematic review of randomized controlled trials. Berlin: Universitätsverlag der TU Berlin; 2015.
13. Iribarren SJ, Cato K, Falzon L, Stone PW. What is the economic evidence for mHealth? A systematic review of economic evaluations of mHealth solutions. PloS one. 2017;12(2):e0170581.
14. Mansaray A, Haghparast-Bidgoli H. Economic evidence on mHealth and community mobilisation interventions for the prevention and management of type 2 diabtes: A systematic review. 2017.
15. Balagopal P, Kamalamma N, Patel TG, Misra R. A community-based diabetes prevention and management education program in a rural village in India. Diabetes care. 2008;31(6):1097-104.
16. Balagopal P, Kamalamma N, Patel TG, Misra R. A community-based participatory diabetes prevention and management intervention in rural India using community health workers. The Diabetes educator. 2012;38(6):822-34.
17. Sathish T, Williams ED, Pasricha N, Absetz P, Lorgelly P, Wolfe R, et al. Cluster randomised controlled trial of a peer-led lifestyle intervention program: study protocol for the Kerala diabetes prevention program. BMC public health. 2013;13:1035.
18. Assah FK, Atanga EN, Enoru S, Sobngwi E, Mbanya JC. Community-based peer support significantly improves metabolic control in people with Type 2 diabetes in Yaounde, Cameroon. Diabetic medicine : a journal of the British Diabetic Association. 2015;32(7):886-9.
19. Baumann LC, Frederick N, Betty N, Jospehine E, Agatha N. A demonstration of peer support for Ugandan adults with type 2 diabetes. International journal of behavioral medicine. 2015;22(3):374-83.
20. Prost A, Colbourn T, Seward N, Houweling TAJ, Fottrell E, Lewycka S, et al. Women's groups practising participatory learning and action to improve maternal and newborn health in low-resource settings: A systematic review and meta-analysis. The Lancet. 2013;381(9879):1736-46.
21. Prost A, Colbourn T, Seward N, Azad K, Coomarasamy A, Copas A, et al. Women's groups practising participatory learning and action to improve maternal and

For peer review only - http://bmjopen.bmj.com/site/about/guidelines.xhtml

newborn health in low-resource settings: a systematic review and meta-analysis. The Lancet. 2013;381(9879):1736-46.
22. Ahmed SM, Alam BB, Anwar I, Begum T, Huque R, Khan JAM, et al. Bangladesh Health System Review. Health Systems in Transition. 2015;5(3).
23. Michie S, van Stralen MM, West R. The behaviour change wheel: a new method for characterising and designing behaviour change interventions. Implementation science : IS. 2011;6:42.
24. Michie SJ, M.; Abraham, C.; Lawton, R.; Parker, D.; Walker, A., on behalf of the 'Psychological Theory' Group. Making psychological theory useful for implementing evidence based practice: a consensus approach. Qual Saf Health Care. 2005(14):26-33.
25. Michie S, Richardson M, Johnston M, Abraham C, Francis J, Hardeman W, et al. The behavior change technique taxonomy (v1) of 93 hierarchically clustered techniques: building an international consensus for the reporting of behavior change interventions. Ann Behav Med. 2013;46(1):81-95.
26. Fottrell E, Azad K, Kuddus A, Younes L, Shaha S, Nahar T, et al. The effect of increased coverage of participatory women's groups on neonatal mortality in Bangladesh: a cluster randomized trial. JAMA Pediatrics. 2013;167(9):816-25.
27. Klose T. The contingent valuation method in health care. Health Policy. 1999;47(2):97-123.
28. O'Brien B, Gafni A. When do the "dollars" make sense? Toward a conceptual framework for contingent valuation studies in health care. Med Decis Making. 1996;16(3):288-99.
29. Diener A, O'Brien B, Gafni A. Health care contingent valuation studies: a review and classification of the literature. Health Econ. 1998;7(4):313-26.
30. Hansen KS, Pedrazzoli D, Mbonye A, Clarke S, Cundill B, Magnussen P, et al. Willingness-to-pay for a rapid malaria diagnostic test and artemisinin-based combination therapy from private drug shops in Mukono District, Uganda. Health Policy Plan. 2013;28(2):185-96.
31. Shariful Islam SM, Lechner A, Ferrari U, Seissler J, Holle R, Niessen LW. Mobile phone use and willingness to pay for SMS for diabetes in Bangladesh. J Public Health (Oxf). 2016;38(1):163-9.
32. Russell S, Fox-Rushby J, Arhin D. Willingness and ability to pay for health care: a selection of methods and issues. Health Policy Plan. 1995;10(1):94-101.
33. Byford S, Raftery J. Perspectives in economic evaluation. BMJ. 1998;316(7143):1529-30.
34. Jonsson B. Ten arguments for a societal perspective in the economic evaluation of medical innovations. Eur J Health Econ. 2009;10(4):357-9.
35. Fiedler JL, Villalobos CA, De Mattos AC. An activity-based cost analysis of the Honduras community-based, integrated child care (AIN-C) programme. Health Policy Plan. 2008;23(6):408-27.
36. Johns B, Baltussen R, Hutubessy R. Programme costs in the economic evaluation of health interventions. Cost Eff Resour Alloc. 2003;1(1):1.
37. Batura N, Pulkki-Brannstrom AM, Agrawal P, Bagra A, Haghparast-Bidgoli H, Bozzani F, et al. Collecting and analysing cost data for complex public health trials: reflections on practice. Global health action. 2014;7:23257.
38. Hutton G, Baltussen R. Cost valuation in resource-poor settings. Health Policy Plan. 2005;20(4):252-9.
39. Walker D. Cost and cost-effectiveness guidelines: which ones to use? Health Policy Plan. 2001;16(1):113-21.

For peer review only

40. Conteh L, Walker D. Cost and unit cost calculations using step-down accounting. Health Policy Plan. 2004;19(2):127-35.
41. Gray AM, Clarke PM, Wolstenholme JL, Wordsworth S. Applied Methods of Cost-effectiveness Analysis in Healthcare (Handbooks in Health Economic Evaluation). Oxford Oxford University Press; 2011.
42. Drummond MF, Sculpher MJ, Claxton K, Stoddart GL, Torrance GW. Methods for the economic evaluation of health care programmes: Oxford university press; 2015.
43. NICE. How NICE Measures Value for Money in Relation to Public Health Interventions. London: NICE (http://www.nice.org.uk) 2013.
44. Tan-Torres Edejer T BR, Adam T, Hutubessy R, Acharya A, Evans DB, Murray CJ. Making choices in health: WHO guide to cost-effectiveness analysis. Geneva: World Health Organization; 2003.
45. Claxton K, Revill P, Sculpher M, Wilkinson T, Cairns J, Briggs A. The Gates Reference Case for Economic Evaluation. The Bill and Melinda Gates Foundation; 2014.
46. Husereau D, Drummond M, Petrou S, Carswell C, Moher D, Greenberg D, et al. Consolidated Health Economic Evaluation Reporting Standards (CHEERS)--explanation and elaboration: a report of the ISPOR Health Economic Evaluation Publication Guidelines Good Reporting Practices Task Force. Value Health. 2013;16(2):231-50.
47. Woods B, Revill P, Sculpher M, Claxton K. Country-Level Cost-Effectiveness Thresholds: Initial Estimates and the Need for Further Research. Value Health. 2016;19(8):929-35.
48. Verguet S, Kim JJ, Jamison DT. Extended Cost-Effectiveness Analysis for Health Policy Assessment: A Tutorial. Pharmacoeconomics. 2016;34(9):913-23.

For peer review only

For peer review only

For peer review only - http://bmjopen.bmj.com/site/about/guidelines.xhtml

Table 1: Overview of resource use and costs measures included in the economic evaluation of the D-Magic interventions.

Perspective/cost category		Type of costs	Description	Sources	Sample size
**Provider**	Program/implementing agency	Direct	Costs of implementing mHealth, community mobilisation and HSS interventions	(1) Project accounts of the implementing agencies (2) Interviews with the project staff	N/A
		Indirect	The opportunity cost of volunteer experts attended the mHealth design meetings, donated items, etc.	(1) Project records on numbers of meeting, attendants etc. (2) Published reports on local wage information based on skill category (3) Field offices' inventory information	(1) All meetings held and number of people attended in the meetings will be recorded (2) The list of all equipment in the field offices
	Public healthcare providers	Direct	Changes in utilisation of T2DM and NCD related services at the public health facilities in the study area	(1) Detailed audit and costing study of the health facilities (2) Baseline and end-line cross-sectional surveys (for information on changes in costs of health seeking)	(1) Random sample of health facilities at different levels in both intervention and control areas (2) All participants in the study
		Indirect	The opportunity cost of the time spent by the health care providers attending HSS meetings	(1) Project records on numbers of meeting, attendants etc. (2) Published reports on local wage information based on skill category	All meetings held and number of people attended in the meetings will be recorded
**Participants/households**		Direct	Household expenditure on food and non-food	Household consumption expenditure survey	A random sample of 300 households in the study area
			Costs of health seeking for the participants and their households	Baseline and end-line cross-sectional surveys	All participants in the study
		Indirect	Opportunity cost of participation in the groups	Group participants survey	A random sample of 312 group participants (both male and female)

* HSS: health system strengthening, T2DM: type 2 Diabetes mellitus, NCD: non-communicable diseases

For peer review only - http://bmjopen.bmj.com/site/about/guidelines.xhtml

For peer review only

For peer review only - http://bmjopen.bmj.com/site/about/guidelines.xhtml

CHEERS Tool

Section/item		Item no.	Recommendation	Response
Title and abstract	Title	1	Identify the study as an economic evaluation, or use more specific terms such as ''cost-effectiveness analysis'' and describe the interventions compared.	Yes
	Abstract	2	Provide a structured summary of objectives, perspective, setting, methods (including study design and inputs), results (including base-case and uncertainty analyses), and conclusions.	Yes
Introduction	Background and objectives	3a	Provide an explicit statement of the broader context for the study.	Yes
		3b	Present the study question and its relevance for health policy or practice decisions.	Yes
Methods	Target population and subgroups	4	Describe characteristics of the base-case population and subgroups analyzed including why they were chosen.	Yes
	Setting and location	5	State relevant aspects of the system(s) in which the decision(s) need(s) to be made.	Yes
	Study perspective	6	Describe the perspective of the study and relate this to the costs being evaluated.	Yes
	Comparators	7	Describe the interventions or strategies being compared and state why they were chosen.	Yes
	Time horizon	8	State the time horizon(s) over which costs and consequences are being evaluated and say why appropriate.	Yes
	Discount rate	9	Report the choice of discount rate(s) used for costs and outcomes and say why appropriate.	Yes
	Choice of health outcomes	10	Describe what outcomes were used as the measure(s) of benefit in the evaluation and their relevance for the type of analysis performed.	Yes
	Measurement of effectiveness	11 a)	Single study–based estimates: Describe fully the design features of the single effectiveness study and why the single study was a sufficient source of clinical effectiveness data.	Yes
		11 b)	Synthesis-based estimates: Describe fully the methods used for the identification of included studies and synthesis of clinical effectiveness data.	n/a
	Measurement and valuation of preference based outcomes	12	If applicable, describe the population and methods used to elicit preferences for outcomes.	n/a
	Estimating resources and costs	13 a)	Single study–based economic evaluation: Describe approaches used to estimate resource use associated with the alternative interventions. Describe primary or secondary research methods for valuing each resource item in terms of its unit cost. Describe any adjustments made to approximate to opportunity costs.	Yes
		13 b	Model-based economic evaluation: Describe approaches and data sources used to estimate resource use associated with model health states. Describe primary or secondary research methods for valuing each resource item in terms of its unit cost. Describe any adjustments made to approximate to opportunity costs.	n/a

	Currency, price date, and conversion	14	Report the dates of the estimated resource quantities and unit costs. Describe methods for adjusting estimated unit costs to the year of reported costs if necessary. Describe methods for converting costs into a common currency base and the exchange rate.	Yes
	Choice of model	15	Describe and give reasons for the specific type of decision-analytic model used. Providing a figure to show model structure is strongly recommended.	n/a
	Assumptions	16	Describe all structural or other assumptions underpinning the decision-analytic model.	n/a
	Analytical methods	17	Describe all analytic methods supporting the evaluation. This could include methods for dealing with skewed, missing, or censored data; extrapolation methods; methods for pooling data; approaches to validate or make adjustments (e.g., half-cycle corrections) to a model; and methods for handling population heterogeneity and uncertainty.	Yes
Results	Study parameters	18	Report the values, ranges, references, and if used, probability distributions for all parameters. Report reasons or sources for distributions used to represent uncertainty where appropriate. Providing a table to show the input values is strongly recommended.	Not possible in the protocol although the process is described
	Incremental costs and outcomes	19	For each intervention, report mean values for the main categories of estimated costs and outcomes of interest, as well as mean differences between the comparator groups. If applicable, report incremental cost-effectiveness ratios.	Not possible in the protocol although the process is described
	Characterising uncertainty	20 a	Single study–based economic evaluation: Describe the effects of sampling uncertainty for estimated incremental cost, incremental effectiveness, and incremental cost-effectiveness, together with the impact of methodological assumptions (such as discount rate, study perspective).	Not possible in the protocol although the process is described
		20 b	Model-based economic evaluation: Describe the effects on the results of uncertainty for all input parameters, and uncertainty related to the structure of the model and assumptions.	n/a
	Characterising heterogeneity	21	If applicable, report differences in costs, outcomes, or cost-effectiveness that can be explained by variations between subgroups of patients with different baseline characteristics or other observed variability in effects that are not reducible by more information.	Not possible in the protocol although the process is described
Discussion	Study findings, limitations, generalisability, and current knowledge	22	Summarize key study findings and describe how they support the conclusions reached. Discuss limitations and the generalizability of the findings and how the findings fit with current knowledge	Not possible in the protocol although the process is described
Other	Source of funding	23	Describe how the study was funded and the role of the funder in the identification, design, conduct, and reporting of the analysis. Describe other nonmonetary sources of support.	Yes
	Conflicts of interest	24	Describe any potential for conflict of interest among study contributors in accordance with journal policy. In the absence of a journal policy, we recommend authors comply with International Committee of Medical Journal Editors' recommendations.	Yes
